# Performance of a Novel Electronic Nose for the Detection of Volatile Organic Compounds Relating to Starvation or Human Decomposition Post-Mass Disaster

**DOI:** 10.3390/s24185918

**Published:** 2024-09-12

**Authors:** Emily J. Sunnucks, Bridget Thurn, Amber O. Brown, Wentian Zhang, Taoping Liu, Shari L. Forbes, Steven Su, Maiken Ueland

**Affiliations:** 1 Centre for Forensic Sciences, School of Mathematical and Physical Sciences, University of Technology Sydney, Ultimo, NSW 2007, Australia; emily.j.sunnucks@student.uts.edu.au (E.J.S.); bridget.thurn@student.uts.edu.au (B.T.); amber.brown@alumni.uts.edu.au (A.O.B.); 2 Hyphenated Mass Spectrometry Laboratory, School of Mathematical and Physical Sciences, University of Technology Sydney, Ultimo, NSW 2007, Australia; 3 Faculty of Engineering and Information Technology, University of Technology Sydney, Ultimo, NSW 2007, Australia; wentian.zhang@alumni.uts.edu.au (W.Z.); suweidong@sdfmu.edu.cn (S.S.); 4 College of Artificial Intelligence and Big Data for Medical Sciences, Shandong First Medical University, Jinan 250117, China; 5 Academy of Advanced Interdisciplinary Research, Xidian University, Xi’an 710071, China; taoping.liu@outlook.com; 6 Department of Chemistry and Biochemistry, University of Windsor, Windsor, ON N9B 3P4, Canada; shari.forbes@uwindsor.ca

**Keywords:** sensor, DVI, machine olfaction, metal oxide sensors, decomposition odor, VOCs

## Abstract

There has been a recent increase in the frequency of mass disaster events. Following these events, the rapid location of victims is paramount. Currently, the most reliable search method is scent detection dogs, which use their sense of smell to locate victims accurately and efficiently. Despite their efficacy, they have limited working times, can give false positive responses, and involve high costs. Therefore, alternative methods for detecting volatile compounds are needed, such as using electronic noses (e-noses). An e-nose named the ‘NOS.E’ was developed and has been used successfully to detect VOCs released from human remains in an open-air environment. However, the system’s full capabilities are currently unknown, and therefore, this work aimed to evaluate the NOS.E to determine the efficacy of detection and expected sensor response. This was achieved using analytical standards representative of known human ante-mortem and decomposition VOCs. Standards were air diluted in Tedlar gas sampling bags and sampled using the NOS.E. This study concluded that the e-nose could detect and differentiate a range of VOCs prevalent in ante-mortem and decomposition VOC profiles, with an average LOD of 7.9 ppm, across a range of different chemical classes. The NOS.E was then utilized in a simulated mass disaster scenario using donated human cadavers, where the system showed a significant difference between the known human donor and control samples from day 3 post-mortem. Overall, the NOS.E was advantageous: the system had low detection limits while offering portability, shorter sampling times, and lower costs than dogs and benchtop analytical instruments.

## 1. Introduction

Mass disaster incidents result from a spontaneous event that can originate from natural or unnatural causes and involve large numbers of victims [1]. The frequency of these events is increasing. Natural causes can include events such as earthquakes, tsunamis, hurricanes, flooding, bushfires, and landslides. Typical unnatural causes include building collapses or explosions from terrorist or industrial causes, and aircraft, train, or other vehicle crashes [1,2]. Although most deaths following a mass disaster are immediate and rapid, some are delayed due to causes such as starvation, dehydration, and blood loss, making rapid detection important [3,4]. Current post-disaster search methods include manual searching, imaging, search and rescue (SAR) dogs, and human remains detection (HRD) dogs. Although successful, there are several disadvantages to these methods: searching methods can often be dangerous, time-consuming, and not always reliable, posing both short- and long-term health risks to searchers, other personnel, and SAR or HRD dogs [3,5,6,7]. Detection dogs are currently the most reliable method to search for mass disaster victims as they can cover large areas rapidly and can detect victims where vision or sound is limited [8]. While detection dogs are an effective search tool, these specialized dogs are expensive to train and maintain, can only work for short periods, can give false positive alerts, and are unable to convey information on what they are detecting [8,9].

Both SAR and HRD dogs use volatile organic compounds (VOCs) to track an odor to its source; these are compounds with high vapor pressure that vaporize readily at room temperature. VOCs are responsible for odor, and are detected by the olfactory system, to then be perceived by the brain as odor or scent. VOCs are released into the environment through sweat, blood, urine [10,11,12], and expired air [11,13] in live victims. Specifically, ketones are present in ante-mortem VOC profiles in high abundance due to starvation. During starvation, the body undergoes ketosis, and ketones are synthesized from the use of fatty acids as an alternative food source [10,14,15]. In deceased victims, VOCs are released through the decomposition process from the breakdown of macromolecules and are also produced from microbial activity [5,16,17,18]. Decomposition VOCs from human donors have been studied during a mass disaster simulation; Ueland et al. [5] placed six donors under rubble for one month to simulate a building collapse. The VOC profile was sampled over the month period to determine how the effects of building debris affected the VOC profile. Overall, they identified and investigated sulfur compounds due to their association with decomposition VOC profiles. Specifically, dimethyl disulfide (DMDS) and dimethyl trisulfide (DMTS) are produced as a result of protein breakdown and through microbial activity, and have been found in decomposition VOC profiles from day one post-mortem, making them ideal biomarkers for searching [5,17]. Additionally, alcohols and hydrocarbons were found in high abundance and increasing concentration in the earlier days of the mass disaster simulation.

Current analytical methods for the identification of the VOC profile produced from living or deceased victims are a two-step approach, requiring samples to be collected in-field, and then analyzed in the laboratory using benchtop instrumentation. Currently, two-dimensional gas chromatography coupled with time-of-flight mass spectrometry (GC × GC − TOFMS) is utilized to separate and identify the components of complex VOC profiles [5,9,19,20,21,22]. Despite its high sensitivity, GC × GC − TOFMS has several limitations: the instrument is expensive to buy and maintain, requires user expertise for analysis, is not portable, and has long run times. Therefore, the need for a new field-based system for VOC detection is evident, making electronic nose technology invaluable.

Electronic noses (e-noses) are a gas measurement system which imitate the mammalian olfactory system by collecting and analyzing the VOCs in air samples [23]. E-noses use the combination of a multi-sensor array and pattern recognition to generate a unique ‘chemical fingerprint’ for VOCs [24]. This allows for the detection and differentiation of simple or complex VOC profiles, providing real-time results without the need for user expertise or benchtop instrumentation [25,26,27,28]. E-noses can be fitted with different chemosensors of partial specificity such as metal oxide (MOS), organic CPs, chemocapacitors (CAPs), and gravimetric sensors; each sensor material exhibits different resistance changes when exposed to different VOCs [23,24]. The usage of different materials is dependent on the specific application of the e-nose. E-noses have a wide range of applicability across various industries, where they have been utilized to grade the freshness of food [23,29], to recognize the characteristic odors of cancers and diseases [29,30], drug detection [27,28], or food and alcohol fraud [31,32]. Current chemometric methods used with e-noses for pattern recognition include principal component analysis (PCA) [30,33], which allows visualization of the e-noses’ ability to differentiate different VOCs or VOC profiles. Several limitations with e-noses have been previously highlighted, which are predominantly related to their inability to identify individual VOCs within profiles and quantify compound concentrations. Sensor drift, or the gradual unpredictable changes in sensor response over time, from environmental factors, general use, or sensor poisoning has similarly been recognised as an issue which affects the reproducibility of e-nose results and influences reliability [29,33]. Furthermore, the presence of air patches and eddy currents in the sensor chamber can cause changes in analyte concentration, which have been understood to reflect in sensor response. These changes in sensor response subsequently affect detection limits and cause fluctuations in results [34], posing a challenge when implementing pattern recognition and assessing reliability. Despite these initial drawbacks of the technology, e-nose data interpretation can be improved through machine learning algorithms such as neural networks, which can improve sensor selectivity and recognize complex VOC profiles [35,36,37,38,39].

**Figure 1:**
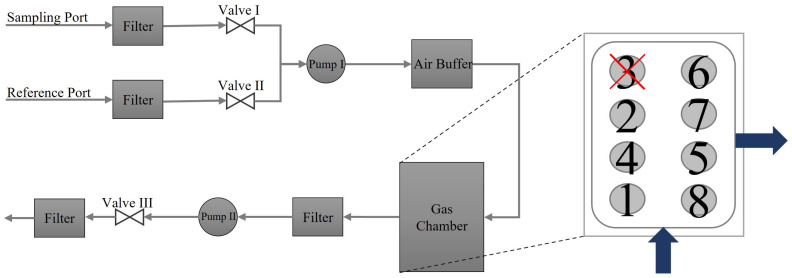
Schematic of the configuration of the NOS.E air intake system and sensor positioning (sensor numbers 1–8) within the gas chamber; arrows represent the direction of airflow through the system, with the blue arrows highlighting airflow through the sensor chamber. The sensor 3 position was not used in this study.

The NOS.E is an e-nose that has been developed at The University of Technology Sydney (UTS) [9,31,33,40,41,42,43]. The NOS.E contains an array of commercially available metal oxide gas sensors that are used to detect different VOC classes. Specifically, the sensors were chosen to target chemical classes such as alcohols, hydrocarbons, ketones, and sulfur- and nitrogen-containing compounds, which are abundant in the ante-mortem [3,10,11,12,13] and decomposition VOC profiles [16,17]. The sensors are comprised of a metal oxide semiconductor (MOS) layer, where, based on the sensor type and compound classes present, the conductivity of the semiconductor increases with the analyte concentration [44]. The NOS.E system has an automated airflow and control system (Figure 1), which assists in decreasing the distortion of VOC analysis results due to deviations in airflow, since duration and speed have been seen to affect the sensor response [33]. Air flows into the open-designed sensor chamber over the sensors one way through four channels, not flowing directly over the sensors, then exits through a single output (Figure 1). The impact of eddy currents within the sensor chamber on sensor response is unknown, and it is unclear whether these air currents influence the system’s repeatability and detection limits. Additionally, how these air currents affect sensor response in relation to sensor positioning within the sensor chamber is also undetermined.

While the NOS.E cannot provide identification of the individual compounds present within the VOC profile, the pattern of the collective sensor responses provides sufficient information to detect and distinguish between chemical compounds. The NOS.E has previously proven its ability to classify compounds of similar chemical classes in studies where different iterations of the system have been utilized in the differentiation of alcohols and perfumes, or to monitor human breath for illnesses [31,33,40,41,42,43]. Brown et al. [9] determined the system’s in-field performance, monitoring human cadavers in the early decomposition stages; they successfully distinguished between the human decomposition and environmental VOC profiles.

Despite its use in prior studies, the current capabilities and limitations of the NOS.E are unknown. This work aimed to assess the capabilities of the system’s current configuration using chemical standards in a controlled laboratory environment to determine if the system can differentiate ante-mortem and decomposition VOC profiles. Additionally, the system was tested in a mass disaster simulation to determine the in-field applicability of the system with donated human cadavers. While e-noses have demonstrated limited capability in detecting individual components of VOC profiles due to the partial specificity of their gas sensors, this study utilized individual compounds commonly found in ante-mortem and decomposition VOC profiles. These specific analytes were selected to explore the system’s limits while minimizing potential interferences from complex mixtures. Given the dynamic nature and complexity of VOC profiles, replicating and diluting a profile for calibration purposes poses significant challenges. Consequently, this research cannot be used for training pattern recognition or machine learning algorithms, and authentic VOC profiles should be utilized for these purposes.

This research provides the first steps in validating the system as a detection tool with future scope to improve the system’s capabilities through different configurations. Ultimately, the aim of this portable technology is to serve as a search method in the aftermath of mass disasters or in missing persons situations. As a detection tool, the NOS.E can assist in locating and recovering live victims trapped beneath debris, as well as retrieving deceased remains. Additionally, the system can provide searchers with information on the VOC profiles detected, offering an advantage over detection dogs.

## 2. Materials and Methods

### 2.1. Chemicals

Fourteen analytical standards representative of human ante-mortem and decomposition volatile compounds were chosen. The assay percentage (%) of each analyte is as shown in the brackets. Dioctyl ether (99.0), α-terpineol (≥95.0), estragole (≥98.0), bromobenzene (≥99.5), ethylcyclohexane (≥99.0), toluene (99.9), 2-heptanone (≥99.0), 2-pentanone (≥98.0), and dimethyl disulfide (DMDS) (≥98.5) were supplied by Sigma-Aldrich (Macquarie Park, NSW, Australia). Methanol (>99.9), acetone (>99.0), and acetonitrile (ACN) (>99.9) (all HPLC grade) were supplied by Honey Research Chemicals (ChemSupply Australia Pty Ltd., Gillman, SA, Australia). Dimethyl trisulfide (DMTS) (≥98.0) and 4-methylheptane (99.0) from Tokyo Chemical Industry (ChemSupply Australia Pty Ltd., Gillman, SA, Australia) were used.

### 2.2. Sample Preparation

An air dilution method was used to achieve five different VOC concentrations for each analyte; each liquid standard was individually injected into an air-filled sampling bag, and passive diffusion was allowed until the liquid was volatilized. SKC Tedlar Air Sample Bags with dual stainless-steel fittings (Airmet Scientific, Artarmon, NSW, Australia) were filled with compressed air (Industrial grade, Coregas, Yennora, NSW, Australia). The volume of compressed air used to fill each bag was measured using an Allborg mass flow controller GFC (Staton Scientific, Mullumbimby, NSW, Australia), calibrated using an Ellutia 7000 flowmeter (Ellutia Chromatography Solutions, Witchford, Ely, UK) with a filling volume of 500 mL/min. The filling time for each different sampling bag volume (1 L, 10 L, 50 L, and 100 L) was 2 min, 20 min, 100 min, and 200 min, respectively. After filling with air, the analyte was injected into separate sampling bags through the rubber septum fitting, using an eVol XR handheld automated analytical syringe with a SGE 5 μL XCHANGE Syringe (Trajan Scientific Australi Pty Ltd., Ringwood, VIC, Australia). Six different concentrations were produced per analyte in the range of 0.2–119 ppm and were subsequently analyzed using the NOS.E; the final concentrations of each analyte are displayed in Table 1 in parts per million (ppm).

### 2.3. The NOS.E Test Setup

The NOS.E system contains an array of interchangeable metal oxide semiconductor (MOS) sensors, which, in this case, were chosen to target the most abundant chemical classes present in ante-mortem and decomposition VOC profiles. All sensors in the array were manufactured by Figaro Engineering Inc., Mino, Osaka, Japan (Table 2).

The sampling protocol (Table 3) consisted of three phases: pre-conditioning, sampling, and recovery/cleaning. Of the pre-conditioning steps, Chamber Wash I and Vacuum Time I only occurred when the NOS.E was initially switched on and was not repeated during testing. Each sampling protocol consisted of the following steps: Baseline Setup, Vacuum Time II, Sampling Time, Baseline Recovery, and Chamber Wash II. The sampling parameters were chosen based on previous studies [9]; however, it was experimentally determined that a longer chamber washing time was needed, to reduce carryover between samples and to ensure the sensor response returned to baseline. The system was tested with a test mix (methanol, hexane, and DMDS) prior to each testing day, to ensure sensors were functioning correctly and significant sensor drift was not detected. Sensor drift was visually assessed using the dynamic graph within the user interface, NOS.E Analyzer, to detect significant changes in peak height. A sensor was only replaced once it showed minimal or no response to the test mix.

**Figure 2:**
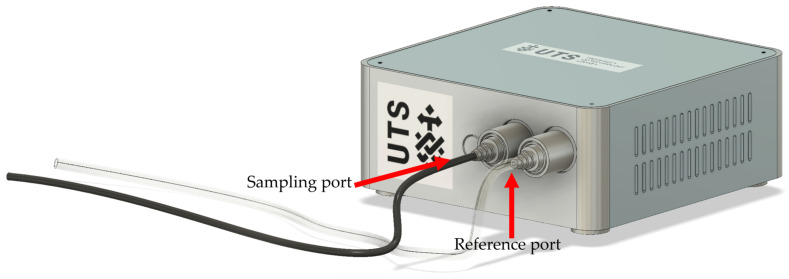
Three-dimensional model of the NOS.E system displaying sampling setup with sampling port, reference port, and tubing.

The setup of the NOS.E used for sample collection was as seen in Figure 2. The reference port was connected to clear, inert Tygon R-3603 tubing (Sigma-Aldrich, Macquarie Park, NSW, Australia), which was fastened to the filling port on the sample bag using a hose clamp to minimize air leakage. The sample port was connected to a Watsford Tubex Flexible Nylon Tube (6 mm OD × 1 mm wall). A 50 L sample bag filled with compressed air was fitted to the reference port and used as reference air, and a second 50 L sample bag filled with compressed air was fitted to the sampling port and used as a blank between different concentrations.

The sampling bags for each concentration and analyte were individually connected to the sampling port, and the sampling bag valve was opened only during the 60 s sampling time. Triplicate repeats of each standard were analyzed for the 10 L, 50 L, and 100 L bags. For the 1 L bags, separate bags were used and analyzed independently since each repeat needed a full liter of air during testing. Three blanks were run between each concentration to mitigate carryover from higher concentration standards.

### 2.4. Experimental Site

A field study was conducted at the Australian Facility for Taphonomic Experimental Research (AFTER; Yarramundi, NSW, Australia). Six donated human cadavers were used for the trial (Table 4). All ethical and legal requirements associated with researching donated human cadavers were acquired through the University of Technology Sydney (UTS) Human Research Ethics Committee (HREC ETH18-2999) and the UTS Body Donation Program with consent provided by donors accordance with the New South Wales (NSW) Anatomy Act (1977). A detailed description of the facility and the environmental conditions at the site is provided in [5]. The simulated disaster was conducted during the Australian summer and remains were allowed to decompose for 14 days post-placement.

The NOS.E was fitted with 5 different sensors (Table 2). A discrete sampling method was carried out by taking replicate (n = 5) samples from pre-determined locations. Samples were taken from an area of the disaster site where remains were present (known) but obstructed from view, and an area of the disaster where no remains were present and at a distance away from any remains (control). Samples were taken on days 1, 3, 5, 7, 9, and 11 post-placement, using the sampling parameters and the NOS.E setup as described in Section 2.3.

### 2.5. Data Analysis

The NOS.E Analyser displayed the sensor response in real time as a dynamic graph, where each sensor was represented by a different color. The data collected by the NOS.E were saved as a text file (.txt) and analyzed using Python programming language (Python 3.10.9) in the Scientific Python Development Environment (Spyder 5.4.1). Sensor responses from baseline setup, test time, and baseline recovery were collected, resulting in 190,000 data points for each sensor. Automated peak detection was performed to determine the response of each sensor, as presented in Brown et al. [9]. In brief, automated peak detection compared the sensor response values to neighboring values until the maxima were determined. Once the maxima were determined, both sides of the peak base were then found and the highest peak base was subtracted from the peak maxima with a prominence value of 0.01. Using Matplotlib (Version 3.7.0), the peak heights were averaged across the three replicates and the standard deviation was calculated.

Matplotlib was used to visualize the data. Sensors that showed zero response to any concentration of standard were excluded (sensors 3 and 8). Outliers were removed from the averaged standard curve data, only in circumstances where significant sensor drift was seen to have occurred, where a concentration had been tested more than 3 months later, and peak height was >0.5 points different from the trendline. Outliers were only removed from sensor TGS 2602, which was seen to have a shorter life than the other sensors; specifically, outliers were removed from sensor 4 for DMDS, 2-heptanone, 2-pentanone, acetone, and estragole, and sensor 7 for dioctyl ether, methanol, ethylcyclohexane, and toluene. These outliers were identified from the 1 μL in the 10 L bag standard since this standard was added at a later stage, after significant sensor drift had occurred. SciPy was used to fit a logarithmic and linear trendline to the data for each sensor; the trendline with the higher determination coefficient was selected. The limit of detection (LOD) was then calculated from the calibration curve for each sensor using the residual standard deviation; the residual standard deviation is the standard deviation of the difference between the observed and predicted values [45], and the equation is displayed below. The LOQ for the system was not established due to the low R^2^ values of the system for most analytes.

$$LOD=\frac{3.3\times s_{residuals}}{slope}$$

To determine the system’s qualitative performance, a PCA plot was created using Python with the Scikit learn and Matplotlib packages. Separate plots were generated for each concentration range (i.e., 0.2 µL of analyte in 10 L of air (Table 1)). The data were centered, and singular value decomposition (SVD) was used to decrease the dimensionality of the data. Individual loadings were calculated for each sensor to display their contribution to the principal component, allowing visualization of the direction and strength of each sensor’s contribution to the principal component.

With respect to the field trial data, the peak responses were exported, and further analysis was completed using MetaboAnalyst (Version 5.0) [46] to perform a partial least squares-discriminant analysis (PLS-DA) and determine feature importance.

## 3. Results

### 3.1. Sensor Responses

Standard curves were generated for each compound to assess how the system responds and performs quantitatively to compounds that are commonly reported in ante-mortem or decomposition VOC profiles, to understand the detection limits of the system and expected sensor response. Sensor response was plotted against the analyte concentration in ppm. The NOS.E was seen to respond to all concentrations of compounds tested (Table 1), except for the lowest concentration of dioctyl ether and toluene (0.2 and 0.5 ppm, respectively). The alcohols, hydrocarbons, ketones, and sulfur- and nitrogen-containing compound classes generated a standard curve, where sensor response increased with concentration following either a linear or logarithmic model. As displayed in Table 5, the model that was produced was dependent on sensor type and the saturation of some sensors at higher concentrations.

**Figure 3:**
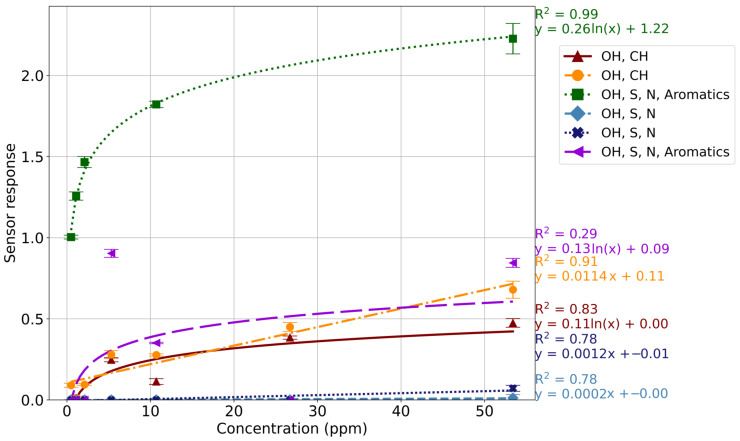
Standard curve of DMDS; sensor response was plotted against concentration (ppm) with each sensor being represented by a different color/marker. The R^2^ value and equation for each sensor are displayed.

Overall, DMDS (Figure 3) had R^2^ values above 0.83 for the OH and CH, and the OH, S, N, and Aromatics sensors, fitting a logarithmic model. Similarly, DMTS (Figure S1) had R^2^ values of >0.70 for the OH and CH (TGS 2610), and the OH, S, N, and Aromatics (TGS 2602) sensors. The R^2^ values across all sensors for both sulfur compounds indicate that the system is unsuitable for quantifying these compounds.

**Figure 4:**
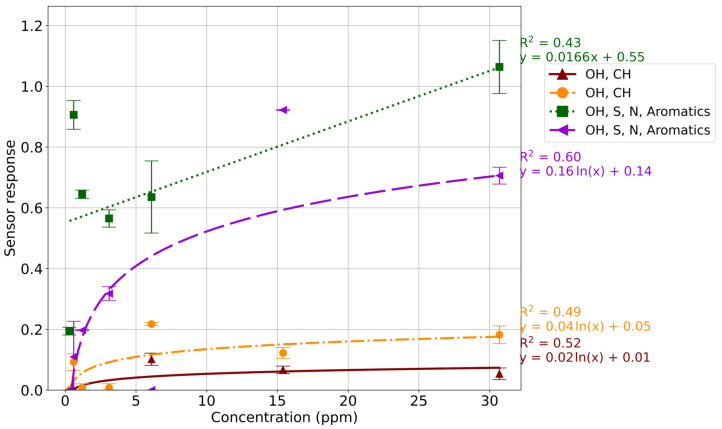
Standard curve of estragole; sensor response was plotted against concentration (ppm) with each sensor being represented by a different color/marker. The R^2^ value and equation for each sensor are displayed.

Sensor response for the detection of ethers and halogens was unpredictable, with estragole (Figure 4) having low R^2^ values for all sensors (<0.60). This was particularly evident from the OH, S, N, and Aromatics sensor (TGS 2602), where sensor response did not correlate with analyte concentration. Similarly, dioctyl ether (Figure S8) and bromobenzene (Figure S7) also produced unpredictable responses in relation to concentration, specifically with the OH, S, N, and Aromatics sensor (TGS 2602). The weak correlation is likely due to the lack of specificity towards these compound classes in the sensor array.

The manufacturer reported the OH, S, N, and Aromatic sensor (TGS 2602) to be sensitive to air contaminants [47] (VOCs, ammonia, H_2_S) and it was seen to respond to all fourteen compounds tested, at all concentrations (Table 6). Similarly, the OH and CH sensors (TGS 2610 and 2600) also responded to all compounds tested, while the OH, S, and N sensors (TGS 2603) were only seen to respond to high concentrations of alcohols, hydrocarbons, halogens, and nitrogen- and sulfur- containing compounds. All sensors produced both logarithmic and linear responses; a logarithmic response was observed when the rate of increase in sensor response was notably higher at lower concentrations, and then plateaued as the concentration increased due to sensor saturation at concentrations > 30 ppm. The linear model was seen in the compounds where the sensors were not saturated at the higher concentrations (Table 5). Each of the OH, S, and N sensors (TGS 2603) were seen to respond to high concentrations of hydrocarbons, alcohols, sulfur, halogens, and nitrogen. The sensors only responded to six out of the fourteen compounds tested and did not respond to the ketones, making this sensor type useful for differentiating between ante-mortem and decomposition VOC profiles.

### 3.2. Limit of Detection

In the context of a mass disaster, the system must have low detection limits since the VOC profile of victims buried under debris needs to be detected. The calculated LOD was between 0.6 and 67.3 ppm for all chemical classes (Table 7). However, experimentally, the system was seen to respond to concentrations lower than the calculated LOD (0.2–1.2 ppm). All compounds but dioctyl ether and toluene responded to the lowest concentration tested. Due to limitations with syringe and sampling bag size, lower concentrations were not tested.

Overall, Sensor 2 (OH, CH sensor; TGS 2600) produced the lowest LOD for most compounds, with sensors 4 and 7 (OH, S, N, Aromatics; TGS 2602) contributing to the lowest LOD for the other compounds. The OH, S, and N (TGS 2603) sensor had the highest detection limits in comparison to the other sensors (Table 6), and only reacted to the higher concentration standards, having a calculated average LOD of 10.4 and 10.9 ppm. The sensor type contributing to the lowest LOD was seen not to be dependent on compound class. The system could detect ethers at 0.3 ppm; however, the LOD was high in the ethers due to the unpredictability in sensor response.

Detection limits were seen not to be affected by sensor positioning within the sensor chamber (Figure 1). Lower detection limits were seen for the sensors in the centre positions of the sensor chamber (Sensor 2; TGS 2600, Sensors 4 and 7; TGS 2602). Whether these lower detection limits are due to sensor type or their position is unknown; however, the LOD was not statistically different to the sensors in other positions. Sensors 5 and 6 (TGS 2603) produced a similar LOD (Table 6) for all compounds they reacted to, though they were placed on opposite ends of the sensor chamber. Sensor 1 exhibited a detection limit of 2 ppm, and no significant differences were observed to suggest that eddy currents within the sensor chamber influenced the system’s LOD. Sensor 8 (TGS 2612) did not react to any compounds; it is unknown whether this is due to the positioning within the sensor chamber and issues with airflow or the specific analytes as this sensor was not trialled in a different position.

### 3.3. Compound Detection

**Figure 5:**
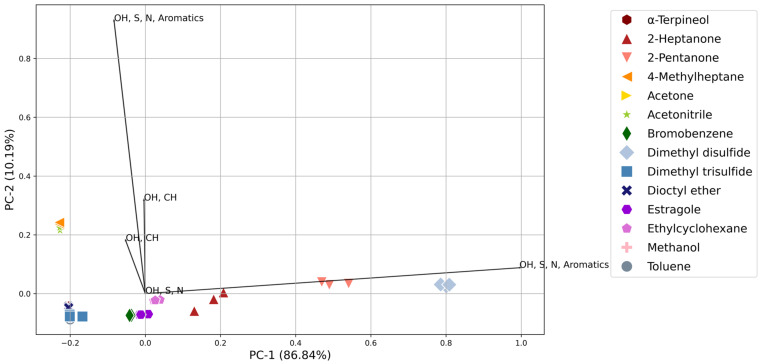
PCA biplot of the standards in the 0.2–1.2 ppm range displaying the separation and clustering of all analytes and the contribution of each sensor type to the principal component.

**Figure 6:**
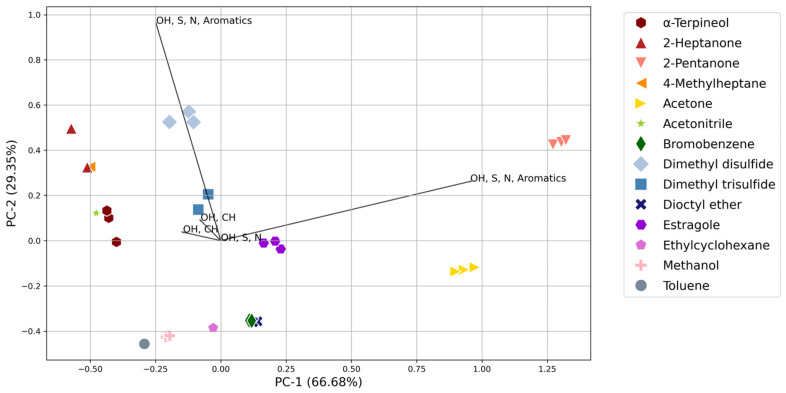
PCA biplot of the standards in the 1.6–11.9 ppm range displaying the separation and clustering of all analytes and the contribution of each sensor type to the principal component.

The collective sensor response of each analyte was assessed to determine the system’s ability to qualitatively separate compounds within similar or distinct compound classes at different concentration ranges. Compounds were grouped based on the volume of analyte added to the volume of air, i.e., all samples using 0.2 µL of analyte in 50 L of air was analyzed together. Principal component analysis (PCA) was used to visualize the collective sensor response of the analytes within a concentration range and to determine if ante-mortem and decomposition VOCs could be differentiated. The triplicate sensor responses for each analyte were plotted in two dimensions, and a PCA biplot was utilized to determine the effect of sensor type on the grouping and separations (Figure 5 and Figure 6). The length of the line indicates the contribution of each sensor type to the principal component.

The NOS.E could successfully distinguish the alkanes, ketones, and sulfur- and nitrogen-containing compounds from each other. Figure 5 and Figure 6 indicate that separation of the compounds was driven by the alcohol, sulfur, nitrogen, and aromatic sensor (TGS 2602) for all concentrations tested. The separation of similar compounds shows promise for the system as a search tool in a mass disaster, where the system could use pattern recognition and machine learning to distinguish between a live or deceased victim, helping searchers when triaging the victims.

The lowest concentration range analyzed (0.2–1.2 ppm) demonstrated good reproducibility between replicates; all replicates were clustered tightly and had a low standard deviation (Figure 5). However, some analytes were found to cluster together, namely, α-terpineol, dioctyl ether, DMTS, and toluene. Similarly, the NOS.E had difficulty distinguishing between all the compounds at the highest concentration analyzed (16–118 ppm) with α-terpineol, 4-methylheptane, acetonitrile, bromobenzene, dioctyl ether, ethylcyclohexane, and methanol all showing poor separation and clustering at this concentration (Figure S13b). Clustering was due to several sensors becoming saturated at this concentration and producing a response lower than the 8–59 ppm concentrations (Figure S16), skewing the PCA results. Furthermore, the ethers and halogenated compounds showed poor differentiation across all concentration ranges, which is likely due to the lack of ether- and halogen-specific sensors within the array (Figure 5, Figure 6, Figures S13–S15). When challenged with the combination of all low and high concentrations of the analytes (0.2–5 ppm and 10–110 ppm; Figures S17 and S18), the system showed separation between the ketones and sulfur-containing compounds, while the rest of the analytes were seen to cluster together in the direction of the two OH, S, N, and Aromatics sensors.

The 1.6–11.9 ppm range was the concentration analyzed that provided the best level of discrimination between the analytes (Figure 6). The NOS.E had a significant sensor response to all compounds at this concentration range. Three or more sensors reacted for each compound, meaning the system’s ability to distinguish different compounds increases with the number of sensors reacting; a greater sensor response and better discrimination facilitate the creation of a more accurate ‘chemical fingerprint’ [26]. All replicate samples for each compound were tightly clustered, validating the NOS.E’s reproducibility of replicates and ability to separate VOCs. Furthermore, compounds from the same chemical class, alcohols, ketones, and sulfur- and nitrogen-containing compounds could be differentiated from each other, which is important in the context of the proposed application of the system.

### 3.4. Field Trial

**Figure 7:**
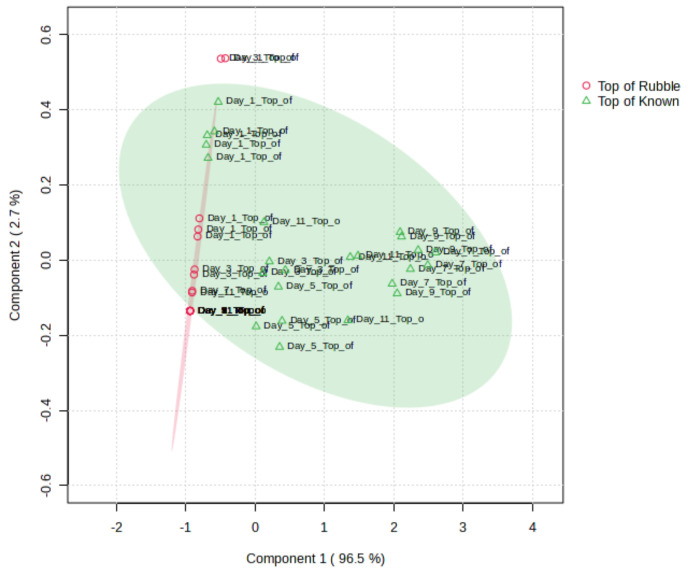
PLS-DA scores plot for the comparison between the sensor response produced from the control (red) and known (green) samples for each sampling day and replicate.

The NOS.E was used in a simulated mass disaster scenario where six human cadavers were placed under rubble, and samples were taken from areas of the disaster site where remains were present (known) and areas of the disaster site where there were no remains (control). A t-test (95% confidence interval) demonstrated significant differences between the known and control sensor responses for all five sensors tested. On days 1 and 3, the samples were clustering close to the control samples on the PLS-DA scores plot, showing that there were limited differences between the control and known sites (Figure 7). The last sampling day, day 11, was clustered with the samples from days 3 and 5, indicating that the VOC profile was similar to the earlier days.

**Figure 8:**
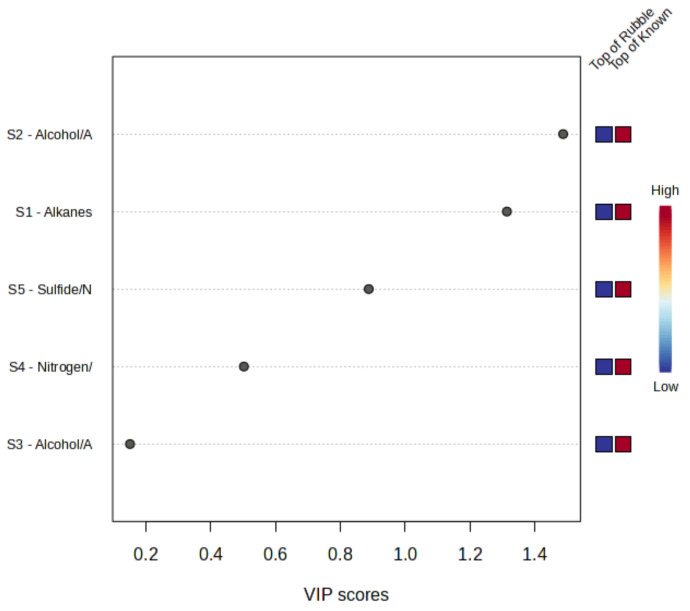
PLS-DA feature importance for each sensor type used in the field trial.

As observed in Figure 8, Sensor 2 (TGS 2602), the OH, S, N, Aromatics sensor, was seen to be the most important feature with PLS-DA analysis, followed by Sensor 1 (TGS 2610) and Sensor 5 (TGS 2603), with both Sensor 2 and 1 having variable importance in a projection (VIP) score > 1. Thus, these sensors are considered significant in the classification of the known samples.

## 4. Discussion

### 4.1. Analytes

Fourteen analytes (Table 1) were chosen to determine the detection capabilities and expected response of the NOS.E for use as a victim detection tool post-mass disasters and to determine the ability of the system to differentiate ante-mortem and decomposition VOC profiles. The compounds tested are significant within the ante-mortem or decomposition VOC profile, with some compounds identified in both VOC profiles. Bromobenzene was the only compound that was not identified in either VOC profile but was included to test how the NOS.E responded to halogens, since halogens have been identified in the decomposition VOC profile [17]. Sulfur-containing compounds are some of the most widely reported decomposition VOCs and are often described as having a ‘rotten egg’ odor, making them an ideal VOC marker following a mass disaster, or to look for human remains in a missing person’s context. Consequently, DMDS and DMTS were analyzed with the e-nose. These compounds are often found in high abundance during the decomposition process and are produced through protein breakdown and microbial activity [9,17,19,48]. Additionally, they were some of the most abundant compounds identified during a simulated mass disaster study [5].

Two alcohols were analyzed: methanol, a simple alcohol, and α-terpineol, a terpene alcohol. Methanol has been identified in several ante-mortem matrices, including expired air [13,49], saliva, blood, feces, and urine [50], while α-terpineol has been identified as a volatile component of urine [51] and was identified during the aforementioned simulated human mass disaster scenario [5]. 2-Heptanone, 2-pentanone, and acetone were also analyzed using the NOS.E; these compounds were chosen due to their prevalence within ante- and decomposition VOC studies and their association with starvation [14,52,53]. As the body experiences starvation, it undergoes ketosis, whereby ketones are synthesised from fatty acids as an alternative fuel source [10,14]. Because ketones are so abundant in the ante-mortem VOC profile due to starvation, this makes them ideal biomarkers for finding live victims in a post-mass disaster context [11]. Acetone is an important compound within the ante-mortem VOC profile since it is a major volatile of breath, blood, sweat, and urine [3,11], and is produced in high concentrations during ketosis, with one study observing a 30-fold increase during starvation [14]. Acetonitrile (ACN) was the only nitrogen-containing compound that was tested and it can be identified within feces, saliva, and the early decomposition VOC profile [50,54].

Estragole and dioctyl ether are ethers that have been identified in the ante-mortem VOC profile and can be found in saliva and feces [50]. They were included to determine the response of the NOS.E to ethers. Finally, toluene, 4-methylheptane, and ethylcyclohexane were analyzed as hydrocarbons are present in high abundance within the ante-mortem and decomposition VOC profiles [21,55,56]. Hydrocarbons were the most detected compounds identified in the first ten days of the mass disaster simulation [5]; consequently, it is important that the system can detect low concentrations of this compound class. Toluene is an aromatic hydrocarbon which has been identified in both the ante-mortem [50,53] and early decomposition VOC profiles [17], while 4-methylheptane and ethylcyclohexane have both been identified in expired air [50,57]. Each compound analyzed differed in molecular weight, vapor pressure, and polarity to determine how the NOS.E reacts to a range of chemically diverse compounds.

### 4.2. Sensor Responses

Sensor type was chosen based on current knowledge about the chemical classes abundant in decomposition and ante-mortem VOC profiles, aligning the system as a tool which searchers can utilize to look for victims following a mass disaster incident. The manufacturer reported that each MOS sensor responds to different compounds [47,58,59,60,61], although it was observed throughout the study that the sensors responded to a broader range of compounds than reported (Table 5). Largely, at least three sensors responded for each compound, facilitating compound detection and allowing differentiation, which will prove beneficial when later employing pattern recognition and machine learning techniques. All sensors were seen to react to at least half of the analytes, excluding the alkane sensor (TGS 2612) [60], which did not respond to any of the compounds tested. The use of this sensor within these VOC profiles should be tested with more concentrated standards or reassessed for use in this context. The OH, S, N, and Aromatic sensor (TGS 2602) was reported by the manufacturer to be sensitive to air contaminants (VOCs, ammonia, H2S) and responded to all analytes. TGS 2602 was also seen to drive differentiation between analytes on PCA analysis, providing the highest variability (Section 3.3). Similarly, the two OH and CH sensors (TGS 2610 and TGS 2600) also responded to all compound classes, although they were only reported by the manufacturer to react to alcohols and alkanes [58,59], while the OH, S, and N sensor (TGS 2603) only responded to alcohols, halogens, and sulfur- and nitrogen-containing compounds, which is consistent with the manufacturer’s specifications. All sensors produced a logarithmic or linear model; this was observed not to be dependent on sensor type. A logarithmic model was produced when sensor saturation occurred at the higher concentrations (Figure S13), while a linear model was produced for the compounds where saturation did not occur and appeared to be compound-specific rather than class-specific. TGS 2603 was the only sensor that consistently followed a linear model due to sensor response only occurring at higher concentrations (>23 ppm). TGS 2602 was seen to be most affected by the effects of sensor drift and had a shorter life compared to otherss. Several outliers were removed due to significant sensor drift from this sensor, predominantly in the concentrations in the 8–59 ppm range, which were analyzed three months later than the other concentrations.

While the purpose of the system is not to specifically quantitate compounds, correlation was observed between concentration and sensor response. However, the R^2^ values were <95 for most compounds, indicating that the linear or logarithmic models cannot be relied on for accurate quantitation. The alcohols (Figures S1 and S11), hydrocarbons (Figures S4, S10 and S12), and sulfur- (Figure 3 and Figure S9) and nitrogen- (Figure S6) containing compounds had R^2^ values of >0.80 for most sensors. The sulfur compounds are some of the most important VOCs within the decomposition VOC profile, and are associated in the literature with being biomarkers for decomposition [5,17,19,48]. This emphasizes the importance of the system’s ability to detect these compounds, especially at low concentrations. The NOS.E was seen to produce a high response to low concentrations of both DMDS (Figure 3) and DMTS (Figure S9), showing promise for the use of the system as a search tool following a mass disaster and to detect human decomposition in other scenarios. The system showed an indeterminate response to the ethers and halogens, with R^2^ values of <0.50 for ethers and <0.66 for the halogenated compound, as a result of the lack of specificity for these compound classes in the sensor array.

There are currently few studies in which electronic nose technology has been examined quantitatively. Sorocki et al. [62] used standard curves to determine the performance of their handheld electronic nose with acetone. However, although they reported using a metal oxide-type sensor, the specific sensor type was not provided [62]. Similarly to the NOS.E, the sensor response from their system was logarithmic, with high R^2^ values for all their sensors in the range of 2–20 ppm. However, sensor response was determined using a different calculation: they used a resistant ratio (R_S_/R_0_) calculation to determine sensor response which corrected for temperature and humidity, since sensors have been seen to be affected by these environmental factors [62]. The NOS.E accounts for these factors by determining a baseline immediately prior to testing, during the baseline setup phase of the sampling procedure. Consequently, any sensor response from these variables should be insignificant. Hence, a temperature variable in post-processing is unnecessary for the NOS.E. Additionally, Peters et al. [26] tested a commercially available handheld e-nose assessing the response of the system quantitatively to toluene. The e-nose utilised was equipped with 16 carbon nanotube (CNT) sensors. The e-nose produced a linear trendline with a high R^2^ value (0.99), whereby the authors used a percentage sensor response calculation, determined using the sensor resistance ratio to determine the overall response. Similarly to the NOS.E, the baseline sensor response under ambient conditions was used in the calculation to eliminate temperature and humidity variables from influencing the results.

While the NOS.E provides some level of quantitation, the R^2^ values were too low to quantify unknown concentrations or determine a LOQ. The results should be improved through the implementation of algorithms to compensate for sensor drift and sensor fluctuations to increase the reliability and accuracy of the system.

### 4.3. Limit of Detection

In a mass disaster scenario where the NOS.E would be deployed to detect the VOC profile of ante- or post-mortem victims, low detection limits across all chemical classes become highly advantageous. The NOS.E had an average LOD of 7.9 ppm, with most compounds having a calculated LOD between 0.6 and 3 ppm, which was not dependent on the class, molecular weight, polarity, or vapor pressure of the analyte. Higher LODs were present in compounds with poor vaporisation properties (α-terpineol; m.p. 31–35 °C [63]) or compound classes where sensors lack specificity (ethers; dioctyl ether and estragole). Experimentally, the system was seen to respond to all compounds between 0.5 and 1 ppm, which is comparable to commercially available e-noses. Peters et al. [26] estimated the LODs for all their compounds rather than calculating them; their estimated LODs were 0.2 ppm for toluene and methylamine and 0.5 ppm for ethyl acetate and acetonitrile [26]. Another handheld analyser was seen to detect acetone concentrations of 2 ppm; this study also estimated the LOD from the lowest concentration instead of determining it statistically [62].

The NOS.E is currently not as sensitive as existing methods for VOC detection. Detection dogs have been reported to detect VOC profiles at lower concentrations, ranging from ppb to parts per trillion (ppt) [64], and analytical instruments can identify compounds at the ppt level [65]. Although detection limits are higher, the NOS.E presents distinct advantages over these methods since it can provide more information than detection dogs while also offering portability and ease of use in comparison to benchtop instruments; however, at its current sensitivity, the system may encounter difficulties when detecting victims below rubble. Therefore, to increase the sensitivity of the system to be more comparable to detection dogs, the sensitivity should be increased by using different sensor materials [66].

The OH and CH sensor (TGS 2600) reacted to all compounds and was responsible for the lowest LOD for most compounds, with the sensor detecting all compound classes in the range of 1.3–21.5 ppm. The manufacturer reported TGS 2600 to have detection limits of 1–30 ppm for H_2_ [58], reflecting what was observed in the sensor response and the LOD values. The OH, S, N, and Aromatics sensor (TGS 2602) had a calculated LOD of 0.6 ppm, confirming the reported detection range of the manufacturer of 1–30 ppm of ethanol or 0.1 ppm of hydrogen sulfide [47]. The OH, S, and N (TGS 2603) sensor had high detection limits compared to other sensors, only reacting to the more concentrated standards, and a calculated LOD 10 times higher than the other sensors.

Generally, eddy currents have been seen to affect the response of sensors, leading to variations in response attributed to airflow dynamics within the chamber [34]. The sensor chamber is an open design, allowing air to mix within the chamber and be distributed to all sensors. Air flows over the sensors one way, through four channels, which do not flow directly over the sensors, then exits through a single output. Additionally, the air intake design of the NOS.E is modelled from the human olfactory system and is a critical element of the e-nose system [33]; it is automated to ensure airflow parameters are kept constant throughout all tests. This study did not observe these air currents as significant since most concentrations had a low standard deviation and tight clustering on PCA (Figure 5 and Figure 6). Sensor positioning within the chamber (Figure 1) did not appear to have a substantial impact on the detection limits, although the sensors positioned within the center of the chamber had the lowest detection limits (Table S1). However, a comparable detection limit was seen for the other sensors. Additionally, Sensors 5 and 6 (TGS 2603) were seen to have the same LOD for the analytes to which they responded, despite these sensors being placed on opposite ends of the sensor chamber and subjected to differing air currents.

The detection limits of the NOS.E were established within a controlled laboratory environment, where the VOC source was connected directly to the tubing. When used within the field for searching, the system will not be directly exposed to the VOC source and will be affected by the wind and other environmental factors, which have been previously understood to affect the system [9]. When used in the field, the system will need to be able to detect individuals trapped below debris and rubble while mounted on a drone or robot to reduce the number of searchers needed in the field, making searching safer. Therefore, lower detection limits are essential, and the system should be improved through different chamber designs or sensor materials [66].

### 4.4. Compound Detection

Analyte differentiation was tested to determine if the system could separate ante-mortem and decomposition compounds. Differentiating these compounds would offer an advantage for searchers over detection dogs. Currently, different types of detection dogs (SAR and HRD dogs) are deployed due to the change in VOC profile from life to death [3,5,10,19]. E-nose technology can detect both, and with the implementation of pattern recognition and machine learning algorithms, can offer more information to searchers.

The system showed effective separation between most analytes at all concentration ranges and could differentiate the compounds of the same functional groups. The 1.6–11.9 ppm range was the lowest concentration that produced the best separation (Figure 6), while the 8–59 ppm range was the only concentration range in which all analytes were differentiated from each other due to the increased number of sensors responding. The NOS.E could successfully distinguish the alkanes, ketones, and sulfur- and nitrogen-containing compounds from each other. This is significant since alkanes, ketones, and sulfur-containing compounds are compound classes that are important within the ante-mortem and early decomposition VOC profiles (Section 4.1). Hence, the NOS.E must be able to detect, recognize, and distinguish these compounds at low concentrations to provide valuable information as a search tool during mass disasters. Separation was limited at the lowest concentration (0.2–1.2 ppm range; Figure 5), which was driven by analytes that produced an insignificant or zero response, or compounds where only one sensor responded, predominantly the OH, S, N, and Aromatics sensor (TGS 2602), demonstrating a lack of sufficient information for discrimination. TGS 2602 was seen to drive differentiation throughout all chemical classes and concentrations.

Differentiation was observed when all the high and low concentration standards were plotted on the same PCA (0.2–5 ppm and 10–110 ppm; Figures S17 and S18), showing promise for the system when having to differentiate between an ante-mortem or decomposition VOC profile. The different concentrations of the same analytes were separated, meaning the system did not identify these as the same compound. However, this is not the purpose of the system since it is expected to provide enough information to provide a detect/no detect to searchers for mass disaster victims. TGS 2602 was seen to drive separation, with the other analytes clustering in the direction of these sensors.

At the 0.2–1.2 ppm concentration range, the specific ante-mortem compounds, ketones, and decomposition compounds, DMDS and DMTS, were differentiated from each other, showing promise for the system when faced with differentiating the two VOC profiles at low concentrations. Brown et al. [9] also reported the same sensor, TGS 2602, as having the highest success in differentiating the various stages of decomposition in an open-air environment, in addition to the OH and CH sensor (TGS 2600) and the OH, S, and N sensor (TGS 2603). Comparatively, the NOS.E had difficulty distinguishing between the compounds at the highest concentration analyzed (16–118 ppm), with toluene, methanol, dioctyl ether, bromobenzene, and ethylcyclohexane clustering together. Sensors 1 and 2, TGS 2610 and 2600, became saturated at this concentration, producing a response lower than the 8–59 ppm concentrations (Figure S13), skewing the PCA results and decreasing differentiation.

Again, the system had difficulty differentiating ethers and halogenated compounds, which was seen consistently throughout all concentration ranges; the sensor array contained no sensors, which the manufacturer claims are sensitive to these chemical classes. Therefore, the sensor array would need to be changed to improve the differentiation of these compounds. While halogens and ethers have been detected in both ante-mortem and decomposition VOC profiles, they do not represent the most prevalent classes in either profile [5]. While it is important that the system can detect these compounds, their differentiation within the ante-mortem and decomposition VOC profile is not a priority for the e-nose.

Although the NOS.E has promising results when detecting and differentiating specific ante-mortem and decomposition analytes, the system cannot detect individual components of a VOC profile due to the partial selectivity of the MOS sensors, this is however not the system’s objective. Instead, the aim of the NOS.E is to be a user-friendly system that can provide a detect/no detect result to non-specialised users when searching for victims during a mass disaster. Zhu et al. [37] discussed that machine learning algorithms, such as artificial neural networks (ANNs), can be combined with MOS sensor arrays to improve the selectivity and recognise complex VOC mixtures. Similarly, Qiao et al. [36] explored how machine learning can be implemented into different sensing materials to assist with health monitoring. They concluded that ANN algorithms could be combined with sensing materials to allow for more accurate monitoring and real-time alerts [36]. Additionally, Galanga et al. [39] implemented artificial neural networks to measure environmental VOCs, finding that machine learning could identify VOCs and predict concentrations with a higher R^2^ than other statistical methods, while Szulczyński et al. [38] found that a machine learning algorithm combined with e-noses could identify the number of components within a VOC mixture.

Implementing machine learning models, such as AANs, into the NOS.E system would be beneficial for the proposed application of the system, where real-time information could be reported back to untrained/non-specialised users in-field to inform searchers of the individuals’ status (alive or deceased) and location, offering more granular information than detection dogs. As discussed earlier, due to the partial selectivity of the metal oxide sensors, the system cannot identify the individual components of a VOC profile, meaning the system cannot be trained to detect biomarkers of decomposition or ante-mortem VOC profiles. However, the objective is to use the entire VOC profile to detect victims since a single biomarker is likely to be insufficient. Future work for the system includes utilizing machine learning to provide an alert when a sensor response distinctive to human decomposition or an ante-mortem VOC profile is detected. Therefore, the NOS.E would require training on the entire ante-mortem and decomposition VOC profiles at differing stages and with large sample sizes to account for the intrinsic variability between humans.

### 4.5. Field Trial

Samples were taken using the NOS.E from areas of a simulated mass disaster site where there were donated human cadavers and an area where no remains were present. The known VOC profile was found to be statistically different from the control samples. The first stage of decomposition, known as the fresh stage, produces minimal VOCs, while autolysis and putrefaction have been recorded to be the most odorous stages of decomposition, producing predominantly sulfur-containing compounds, alcohols, and hydrocarbons [16,18]. The Day 1 and 3 known samples were clustered close to the control samples, indicating that the VOC profile was similar to the control samples (Figure 7) initially, aligning with the previous literature, where minimal variation in the VOC profile was observed in the in-field monitoring of human cadavers with the NOS.E system for the first 3 days post-placement [9]. Additionally, a previous mass disaster simulation saw minimal change in the VOC profile in the first 4 days post-placement [5]. The Day 5 samples were clustered together, demonstrating that the system identified these VOC profiles as similar, and likely the start of autolysis, while the later post-placement samples (Days 7 and 9) were clustered together, implying the VOC profile from these days was comparable, reflecting what is known about human decomposition and the dynamicity of the VOC profile [5,16,18]. The Day 11 samples were clustered with Days 3 and 5, owing to environmental factors, such as wind or a cooler temperature, influencing the VOC production or the system’s capabilities for detection [9].

All sensors used in the sensor array for the field trial (Table 2) were seen to respond to the known samples, with Sensor 2 (TGS 2602), the OH, S, N, and Aromatics sensor, proving to be the most significant feature according to PLS-DA in the field trial, with a VIP score > 1, validating the standard curve findings, where this sensor was a key factor in driving separation with PCA. A field trial by Brown et al. [9] further validated the importance of Sensor 2 (TGS 2602) in detecting decomposition VOC profiles, as they found this sensor to have the most significant response. Sensor 1 (TGS 2610) was the second most important sensor, while Sensor 5 (TGS 2603) followed, supporting the findings from the standard curve and PCA data, proving these sensors are suitable for the proposed application of the system and showing potential as a search tool when implemented in a mass disaster. Overall, the detection limits of the system should be improved, and trials with a different sensor array should be conducted, aiming to improve the system’s ability to detect both living and deceased victims in the earlier stages of decomposition.

## 5. Conclusions

The NOS.E is an e-nose developed at UTS which uses artificial olfaction to detect the volatile organic compounds (VOCs) emitted by mass disaster victims. Current research has predominantly used this e-nose qualitatively; however, no research exists where the configuration and performance of the system was quantitatively evaluated. Thus, the NOS.E was tested with fourteen ante-mortem and decomposition-related analytical standards to ascertain the system’s expected response and performance.

Standard curves were constructed for each of the fourteen analytes, where six different concentrations were plotted against sensor response. The NOS.E produced both logarithmic and linear trendlines throughout the study, which were dependent on the sensor type, compound class, and whether sensor saturation occurred. The NOS.E could detect VOC concentrations of 0.2–1.2 ppm for all alcohols, hydrocarbons, and sulfur- and nitrogen-containing compounds but had difficulty when detecting ethers and halogens due to the limitations of the sensor array.

The results indicate that the system can qualitatively distinguish compounds from different chemical classes that include alcohols, hydrocarbons, ketones, and sulfur- and nitrogen-containing compounds. This shows promise for the system when searching for human victims and differentiating between ante-mortem and decomposition VOC profiles, providing valuable information to searchers regarding the condition of the victim. The 1.6–11.9 ppm range was the lowest concentration range that showed the best differentiation. However, differentiation was limited due to poor sensor response at the lowest concentration, 0.2–1.2 ppm, and the highest concentration, 16–118 ppm, from sensor saturation, which reduced the system’s ability to differentiate the analytes.

When deployed in-field, the NOS.E was advantageous, with a significant sensor response difference seen between the known human remains and control samples in a simulated mass disaster. The system was seen to differentiate the samples from Day 5 post-placement onwards, showing promise for the system when used in a mass disaster to provide a detect/no detect response to searchers to determine if victims are present.

The NOS.E system exhibits potential as a portable device that utilizes VOCs to detect both live and deceased victims in the aftermath of a mass disaster. However, critical future steps for the system include improving the sensitivity through different sensor materials, and validation of the long-term repeatability and additional field testing are necessary to understand how environmental factors and the circumstances of a mass disaster may impact its performance. Additionally, machine learning algorithms should be implemented to alert searchers in-field on whether a victim is detected.

## Figures and Tables

**Table 1:** Final analytes and concentrations which were tested on the NOS.E, in parts per million (ppm), and the air (L) and analyte volume (µL) used to generate the standards.

Air Volume (L)	1	10	10	10	50	100
Analyte Volume (µL)	0.2	1.0	0.4	0.2	0.2	0.2
α-Terpineol	34.6	17.3	6.9	3.5	0.7	0.3
2-Heptanone	45.2	22.6	9.0	4.5	0.9	0.5
2-Pentanone	29.7	14.8	5.9	3.0	0.6	0.3
4-Methylheptane	65.5	32.8	13.1	6.6	1.3	0.7
Acetone	91.6	45.8	18.3	9.2	1.8	0.9
Acetonitrile	45.7	22.8	9.1	4.6	0.9	0.5
Bromobenzene	16.0	8.0	3.2	1.6	0.3	0.2
DMDS	53.4	26.7	10.7	5.3	1.1	0.5
DMTS	45.6	22.8	9.1	4.6	0.9	0.5
Dioctyl ether	30.7	15.4	6.1	3.1	0.6	0.3
Estragole	33.8	16.9	6.8	3.4	0.7	0.3
Ethylcyclohexane	29.0	14.5	5.8	2.9	0.6	0.3
Methanol	118.8	59.4	23.8	11.9	2.4	1.2
Toluene	45.2	22.6	9.0	4.5	0.9	0.5

**Table 2:** Sensor numbers for the air dilution and field trial studies; sensor type as per the manufacturer and their target chemical class.

Sensor Number (Air Dilution)	Sensor Number (Field Trial)	Sensor Type	Target Chemical Class
1	1	TGS 2610	Alcohols, alkanes, and hydrogen
2	3	TGS 2600	Alcohols, alkanes, and hydrogen
4, 7	2	TGS 2602	Alcohols, hydrogen, sulfur, nitrogen, and toluene
5, 6	5	TGS 2603	Alcohols, hydrogen, sulfur, and nitrogen
8	4	TGS 2612	Alkanes

**Table 3:** The NOS.E sampling parameters.

Parameter	Time (s)	Phase
Chamber Wash I	300	Pre-conditioning
Vacuum Time I	10	
Baseline Setup	60	
Vacuum Time II	10	Sampling
Sampling Time	60	
Baseline Recovery	60	Recovery and cleaning
Chamber Wash II	300	

**Table 4:** Donor information for each donor used in the field trial, including donor number, age, sex, and the cause of death (CoD).

Donor Number	Age	Sex	Cause of Death (CoD)
D1	33	Female	Coroner’s case
D2	94	Female	Aspiration Pneumonia, Cerebral Infarct—Left Posterior, Inferior
D3	88	Female	Bronchiectasis, Aspiration Pneumonia
D4	89	Female	Large Bowel Obstruction, Sigmoid Colon Cancer, Heart Failure
D5	77	Male	Multiple Organ Shutdown, Pancreatic Cancer, Liver Metastases
D6	67	Male	Cardiac arrest due to acute myocardial infarction, Diabetes Mellitus—Type 2, Hypertension

**Table 5:** Summary of the model the sensors followed and the average R^2^ of all sensors which responded for each compound.

Compound	Model	Average R^2^ of All Sensors
α-Terpineol	Log and linear	0.8
2-Heptanone	Log	0.82
2-Pentanone	Log	0.67
4-Methylheptane	Log and linear	0.88
Acetone	Log and linear	0.97
ACN	Log and linear	0.87
Bromobenzene	Log and linear	0.66
Dioctyl ether	Log and linear	0.09
DMDS	Log and linear	0.69
DMTS	Log and linear	0.88
Estragole	Log and linear	0.50
Ethylcyclohexane	Log	0.62
Methanol	Log and linear	0.90
Toluene	Log and linear	0.77

**Table 6:** Lowest LOD for each sensor (ppm); the number of compounds each sensor reacted to (n = 14) and the compound classes to which each sensor reacted.

Sensor Number	Sensor Type	Lowest LOD	Number of Analytes	Classes Reacted to
1	TGS 2610	2.0	14	Alcohols, ethers, halogens, hydrocarbons, ketones, nitrogen, and sulfur
2	TGS 2600	1.3	14	
4	TGS 2602	1.3	11	
7	TGS 2602	0.6	10	
5	TGS 2603	10.4	6	Hydrocarbons, alcohols, sulfur, halogens, nitrogen
6	TGS 2603	10.9	7	

**Table 7:** Lowest LOD for each compound by class and the sensor number and its target chemical class responsible.

Compound	Class	LOD (ppm)	Sensor Number	Target Chemical Class
α-Terpineol	Alcohols	67.3	4	OH, S, N, Aromatics
Methanol		1.6	2	OH, CH
Dioctyl ether	Ethers	10.8	2	OH, CH
Estragole		4.4	7	OH, S, N, Aromatics
Bromobenzene	Halogen	2.4	2	OH, CH
4-Methylheptane	Hydrocarbons	0.6	7	OH, S, N, Aromatics
Ethylcyclohexane		3.0	2	OH, CH
Toluene		2.5	2	OH, CH
2-Heptanone	Ketones	1.3	2	OH, S, N, Aromatics
2-Pentanone		3.6	2	OH, S, N, Aromatics
Acetone		8.0	1	OH, S, N, Aromatics
ACN	Nitrogen-containing	1.9	2	OH, CH
DMDS	Sulfur-containing	2.5	1	OH, S, N, Aromatics
DMTS		1.0	7	OH, S, N, Aromatics

## Data Availability

The data supporting the conclusions of this article will be made available by the authors upon request.

## Supplementary Materials

The following supporting information can be downloaded at: https://www.mdpi.com/article/10.3390/s24185918/s1, Table S1: The sensors which responded to each compound, the target chemical class of the compounds, and the limit of detection model the response best fitted and R^2^ for each compound. Figure S1: Standard curve of α-terpineol; sensor response was plotted against concentration with each sensor being represented by a different color/shape. The R^2^ value and equation for each sensor is displayed. Figure S2: Standard curve of 2-heptanone; sensor response was plotted against concentration with each sensor being represented by a different color/shape. The R^2^ value and equation for each sensor is displayed. Figure S3: Standard curve of 2-pentanone; sensor response was plotted against concentration with each sensor being represented by a different color/shape. The R^2^ value and equation for each sensor is displayed. Figure S4: Standard curve of 4-methylheptane; sensor response was plotted against concentration with each sensor being represented by a different color/shape. The R^2^ value and equation for each sensor is displayed. Figure S5: Standard curve of acetone; sensor response was plotted against concentration with each sensor being represented by a different color/shape. The R^2^ value and equation for each sensor is displayed. Figure S6: Standard curve of acetonitrile; sensor response was plotted against concentration with each sensor being represented by a different color/shape. The R^2^ value and equation for each sensor is displayed. Figure S7: Standard curve of bromobenzene; sensor response was plotted against concentration with each sensor being represented by a different color/shape. The R^2^ value and equation for each sensor is displayed. Figure S8: Standard curve of dioctyl ether; sensor response was plotted against concentration with each sensor being represented by a different color/shape. The R^2^ value and equation for each sensor is displayed. Figure S9: Standard curve of DMTS; sensor response was plotted against concentration with each sensor being represented by a different color/shape. The R^2^ value and equation for each sensor is displayed. Figure S10: Standard curve of ethylcyclohexane; sensor response was plotted against concentration with each sensor being represented by a different color/shape. The R^2^ value and equation for each sensor is displayed. Figure S11: Standard curve of methanol; sensor response was plotted against concentration with each sensor being represented by a different color/shape. The R^2^ value and equation for each sensor is displayed. Figure S12: Standard curve of toluene; sensor response was plotted against concentration with each sensor being represented by a different color/shape. The R^2^ value and equation for each sensor is displayed. Figure S13: (a) Standard curve of methylcyclohexane, toluene and methanol sensor 1 showing the saturation, (b) PCA biplot of the standards in the 16–118 ppm range displaying the separation and clustering of all analytes and the contribution of each sensor type to the principal component. Figure S14: PCA biplot of the standards in the 0.3–2.4 ppm range displaying the separation and clustering of all analytes and the contribution of each sensor type to the principal component. Figure S15: PCA biplot of the standards in the 3.2–23.8 ppm range displaying the separation and clustering of all analytes and the contribution of each sensor type to the principal component. Figure S16: PCA biplot of the standards in the 8–59 ppm range displaying the separation and clustering of all analytes and the contribution of each sensor type to the principal component. Figure S17: PCA biplot of low concentrations of the analytes tested (0.2–5 ppm) displaying the separation and clustering of all analytes and the contribution of each sensor type to the principal component. Figure S18: PCA biplot of high concentrations of the analytes tested (10–110 ppm) displaying the separation and clustering of all analytes and the contribution of each sensor type to the principal component. Figure S19: Box and whisker plot for the distribution of Sensor 1 (TGS 2601) response for the control (top of rubble) vs the known (top of known). Figure S20: Box and whisker plot for the distribution of Sensor 2 (TGS 2602) response for the control (top of rubble) vs the known (top of known). Figure S21: Box and whisker plot for the distribution of Sensor 3 (TGS 2600) response for the control (top of rubble) vs the known (top of known). Figure S22: Box and whisker plot for the distribution of Sensor 4 (TGS 2612) response for the control (top of rubble) vs the known (top of known). Figure S23: Box and whisker plot for the distribution of Sensor 5 (TGS 2603) response for the control (top of rubble) vs the known (top of known). Figure S24: PLS-DA scores plot for the comparison between the sensor response produced from the control (red) and known (green) for each sample taken. Figure S25: PCA biplot of sensor response, displaying the separation and clustering for each sampling day and replicate, and the contribution of each sensor type to the principal component.
