# Supplementary material for: Performance of a Novel Electronic Nose for the Detection of Volatile Organic Compounds Relating to Starvation or Human Decomposition Post-Mass Disaster
Source: Sensors (Basel). 2024 Sep 12;24(18):5918. doi: 10.3390/s24185918 (PMC11435962; doi:10.3390/s24185918)
Supplement: Supplementary file 1 [file sensors-24-05918-s001.zip › sensors-3111615-supplementary.pdf]

Table S1. The sensors which responded to each compound, the target chemical class of the compounds, the limit of detection, model the response best fitted and R^2 for each compound.

Compound	Sensor Number	Target Chemical Class	LOD (ppm)	Model	R^2
α-terpineol	1	OH, CH	104.1	Linear	0.76
	2	OH, CH	92.9	Linear	0.79
	4	OH, S, N, Aromatics	67.3	Linear	0.86
	5	OH, S, N	77.3	Linear	0.78
	6	OH, S, N	78.3	Linear	0.78
	7	OH, S, N, Aromatics	90.5	Log	0.83
2-heptanone	1	OH, CH	6.6	Log	0.41
	2	OH, CH	1.3	Log	0.95
	4	OH, S, N, Aromatics	46.7	Log	1.00
	7	OH, S, N, Aromatics	1.9	Log	0.90
2-pentanone	1	OH, CH	4.3	Log	0.60
	2	OH, CH	3.6	Log	0.68
	4	OH, S, N, Aromatics	87.2	Log	0.82
	7	OH, S, N, Aromatics	4.2	Log	0.61
4-methylheptane	1	OH, CH	2.3	Log	0.85
	2	OH, CH	1.7	Log	0.91
	4	OH, S, N, Aromatics	36.8	Linear	0.78
	7	OH, S, N, Aromatics	0.6	Log	0.99
Acetone	1	OH, CH	8	Linear	0.99
	2	OH, CH	12.5	Linear	0.98
	4	OH, S, N, Aromatics	12.4	Log	0.98
Acetonitrile	1	OH, CH	2.4	Log	0.85
	2	OH, CH	1.9	Log	0.90
	5	OH, S, N	28.9	Linear	0.78
	6	OH, S, N	28.9	Linear	0.97
	7	OH, S, N, Aromatics	26.2	Linear	0.95
Bromobenzene	1	OH, CH	2.7	Log	0.80
	2	OH, CH	2.4	Log	0.83
	4	OH, S, N, Aromatics	137.7	Linear	0.13
	5	OH, S, N	53	Linear	0.78
	6	OH, S, N	53.5	Linear	0.78
DMDS	1	OH, CH	18	Log	0.83
	2	OH, CH	10.8	Linear	0.91
	4	OH, S, N, Aromatics	95.2	Log	0.99
	5	OH, S, N	2.5	Linear	0.78
	6	OH, S, N	20.5	Linear	0.78
DMTS	1	OH, CH	99.9	Linear	0.87
	2	OH, CH	36.5	Linear	0.85
	4	OH, S, N, Aromatics	36.5	Log	0.95
	6	OH, S, N	8.3	Linear	0.78
	7	OH, S, N, Aromatics	21.5	Log	0.98
Dioctyl ether	1	OH, CH	25	Log	0.07
	2	OH, CH	1.3	Log	0.19
	4	OH, S, N, Aromatics	99.7	Linear	0.04

Estragole	1	OH, CH	1	Log	0.52
	2	OH, CH	5.4	Log	0.49
	4	OH, S, N, Aromatics	5.6	Log	0.43
	7	OH, S, N, Aromatics	123.3	Log	0.60
Ethylcyclohexane	1	OH, CH	4.4	Log	0.45
	2	OH, CH	6	Log	0.77
	4	OH, S, N, Aromatics	3	Log	0.02
Methanol	1	OH, CH	2	Log	0.89
	2	OH, CH	1.6	Log	0.93
	4	OH, S, N, Aromatics	13.3	Linear	0.88
	5	OH, S, N	10.4	Linear	0.92
	6	OH, S, N	10.9	Linear	0.91
Toluene	1	OH, CH	3.1	Log	0.75
	2	OH, CH	2.5	Log	0.83
	4	OH, S, N, Aromatics	33.7	Linear	0.72
	5	OH, S, N	98.8	Linear	0.78
	6	OH, S, N	98.8	Linear	0.78

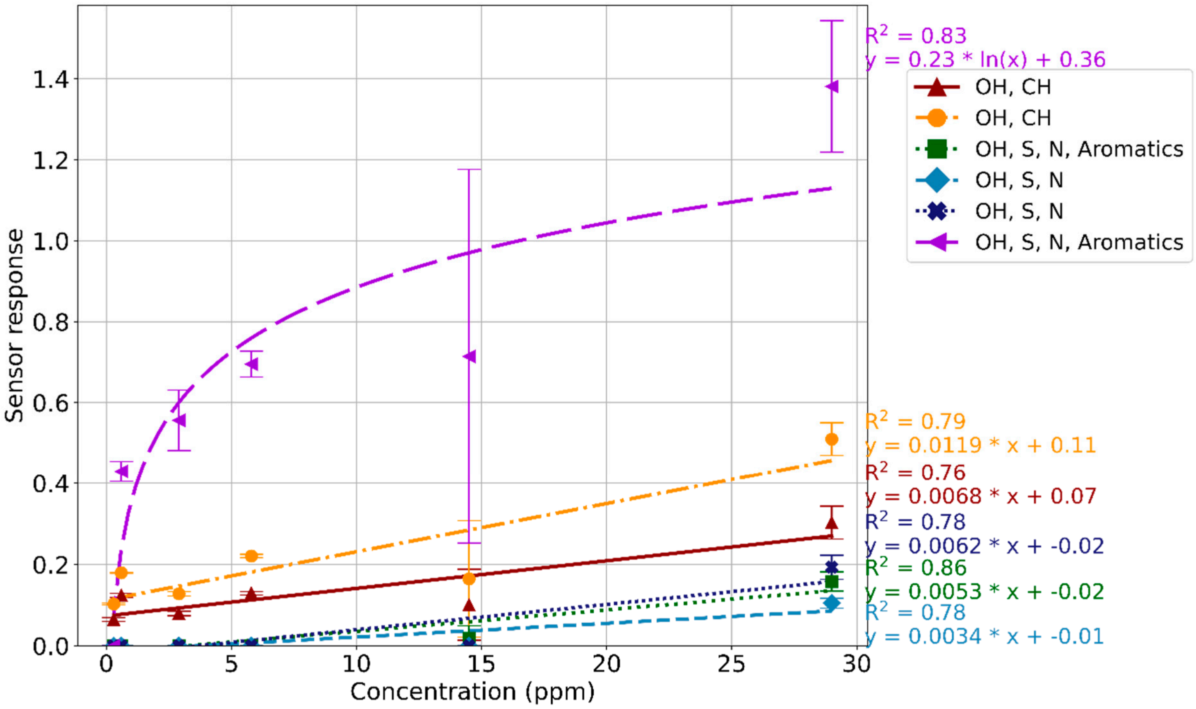

Figure S1: Standard curve of α-terpineol, sensor response was plotted against concentration with each sensor being represented by a different color/ shape. The R^2 value and equation for each sensor is displayed.

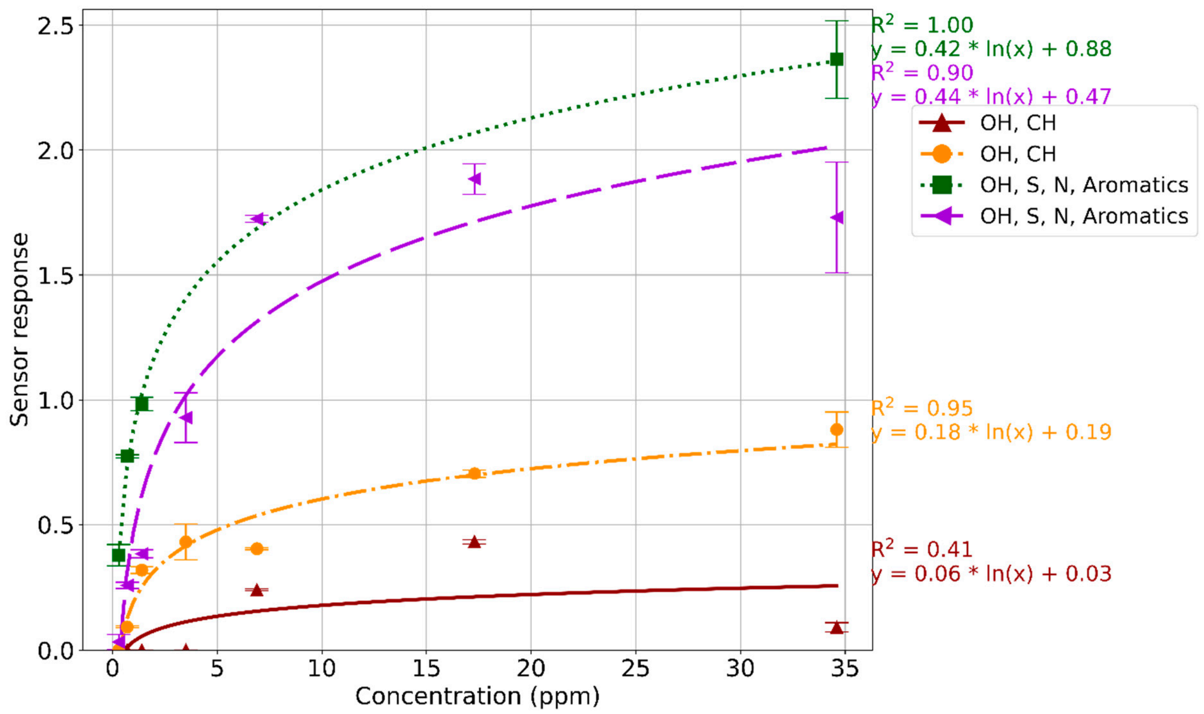

Figure S2: Standard curve of 2-heptanone, sensor response was plotted against concentration with each sensor being represented by a different color/ shape. The R^2 value and equation for each sensor is displayed.

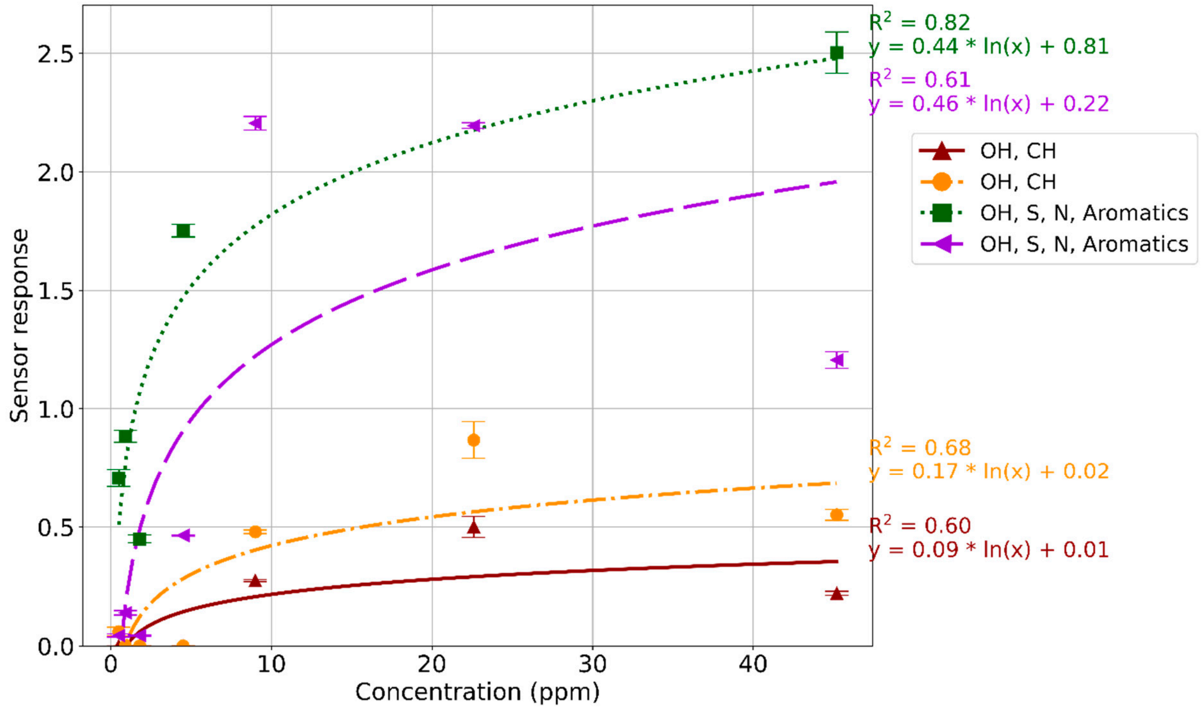

Figure S3: Standard curve of 2-pentanone, sensor response was plotted against concentration with each sensor being represented by a different color/ shape. The R^2 value and equation for each sensor is displayed.

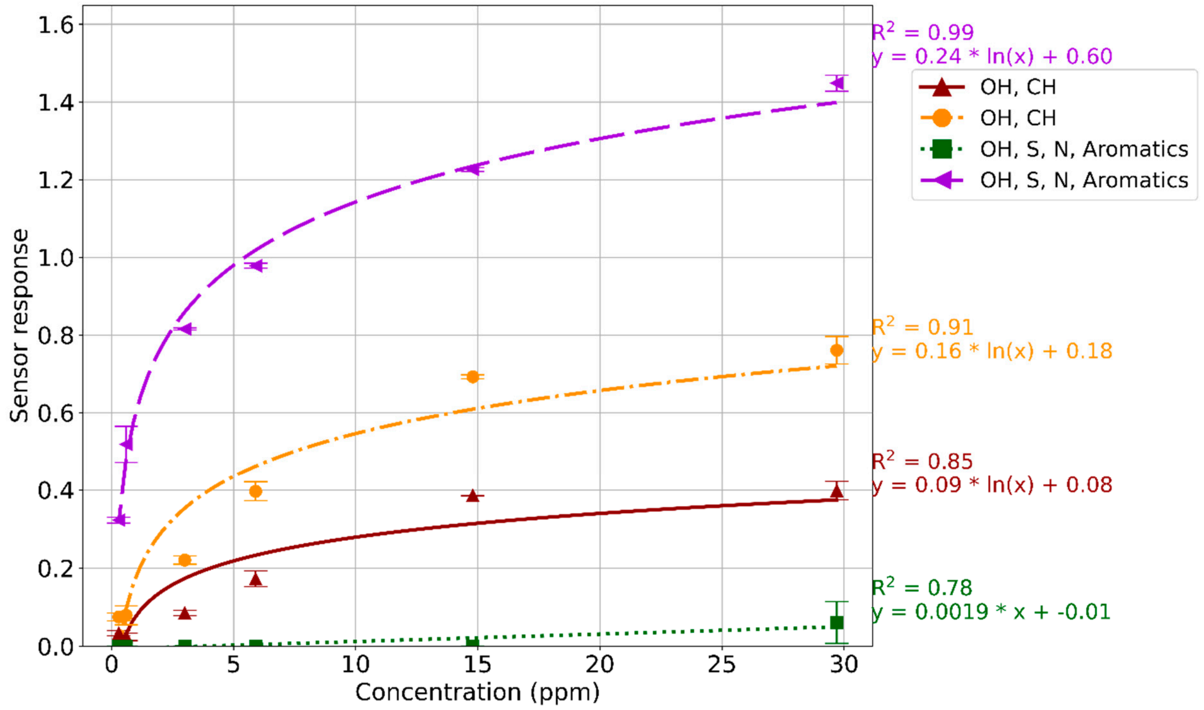

Figure S4: Standard curve of 4-methylheptane, sensor response was plotted against concentration with each sensor being represented by a different color/ shape. The R^2 value and equation for each sensor is displayed.

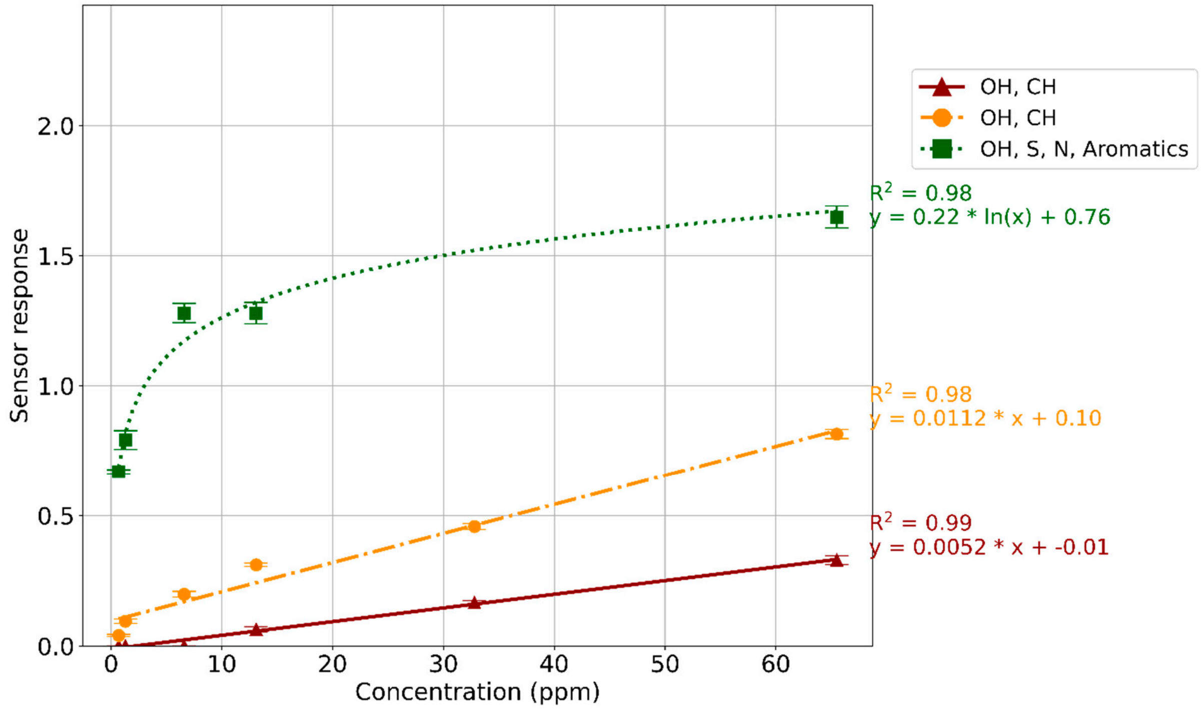

Figure S5: Standard curve of acetone, sensor response was plotted against concentration with each sensor being represented by a different color/ shape. The R^2 value and equation for each sensor is displayed.

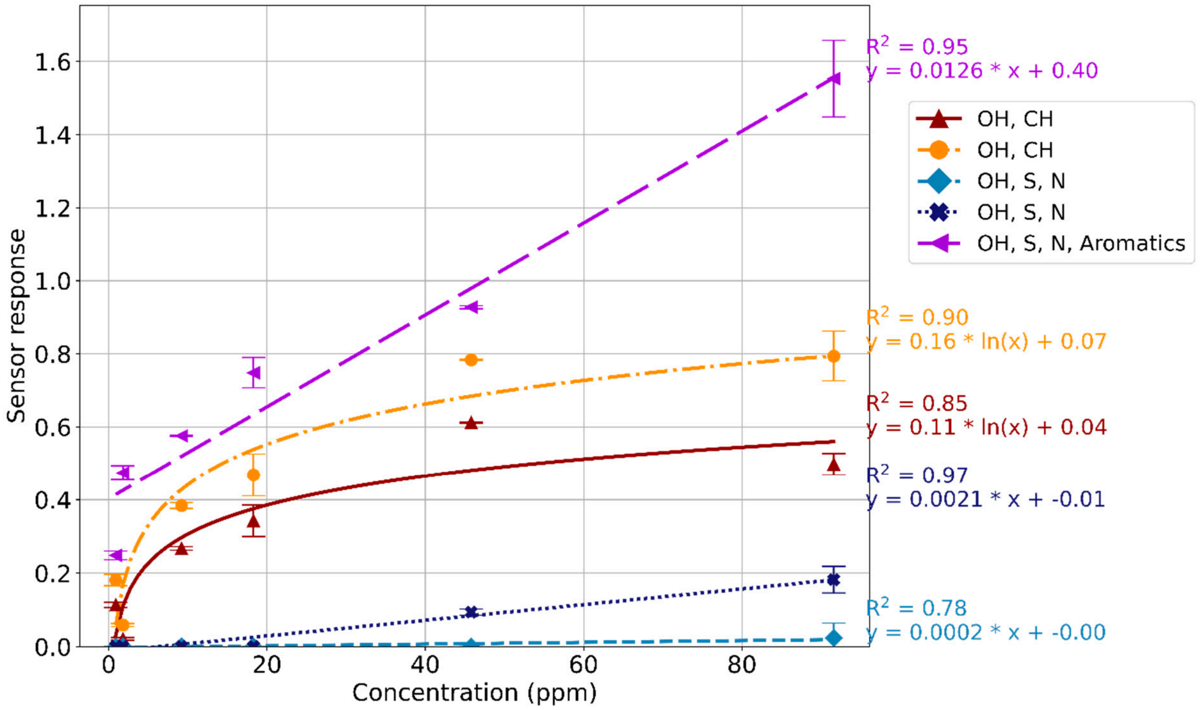

Figure S6: Standard curve of acetonitrile, sensor response was plotted against concentration with each sensor being represented by a different color/ shape. The R^2 value and equation for each sensor is displayed.

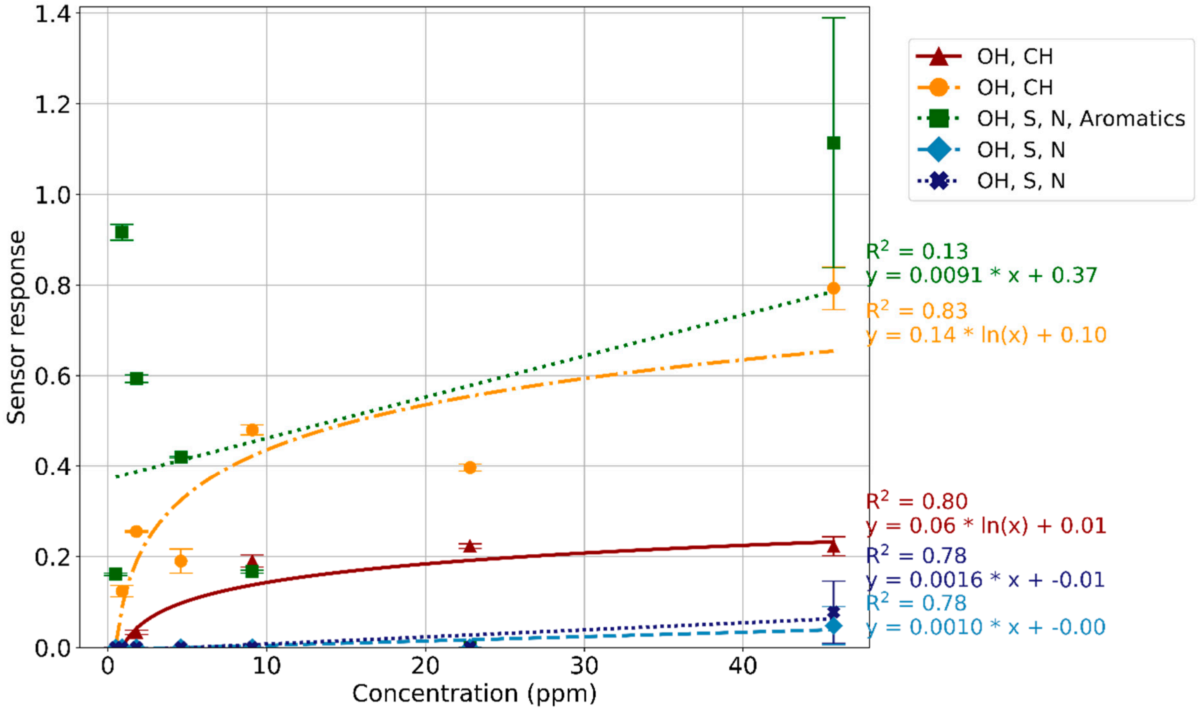

Figure S7: Standard curve of bromobenzene, sensor response was plotted against concentration with each sensor being represented by a different color/ shape. The R^2 value and equation for each sensor is displayed.

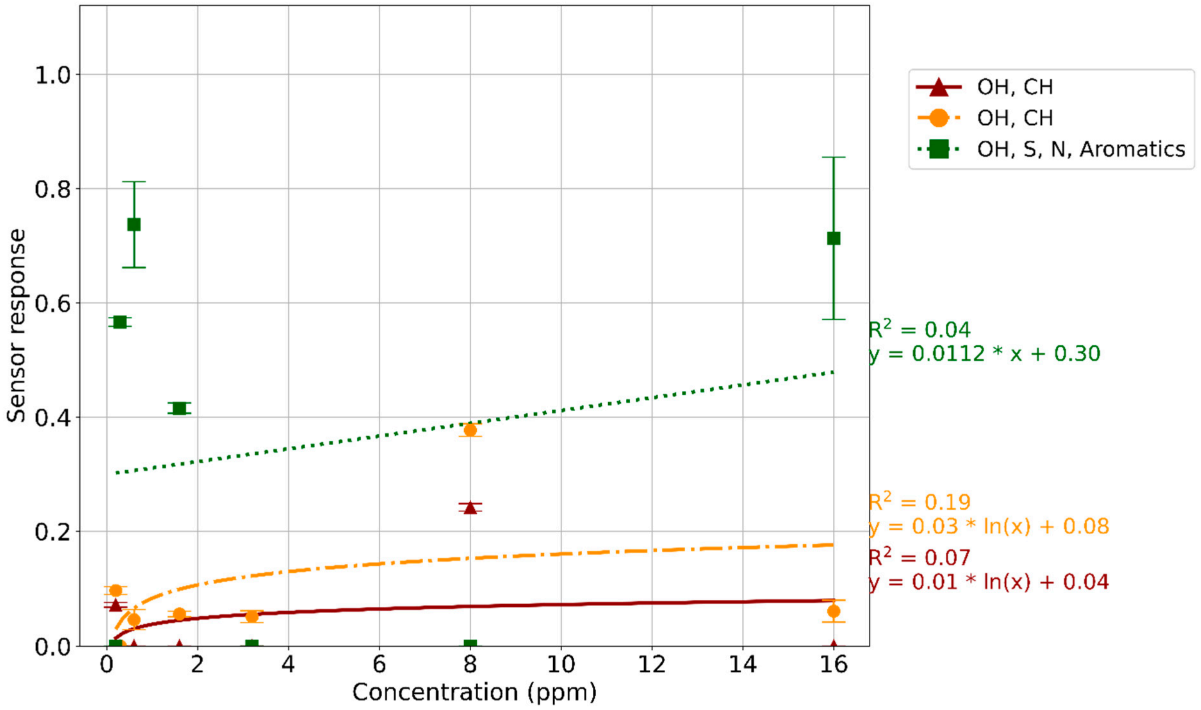

Figure S8: Standard curve of dioctyl ether, sensor response was plotted against concentration with each sensor being represented by a different color/ shape. The R^2 value and equation for each sensor is displayed.

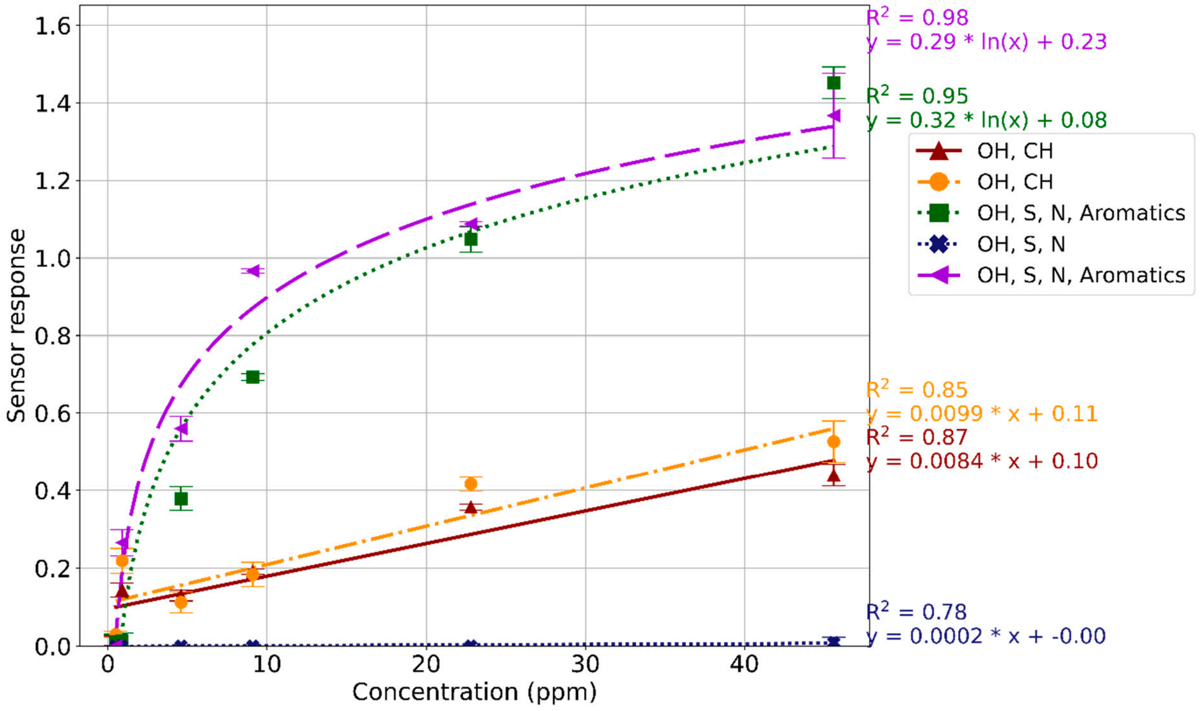

Figure S9: Standard curve of DMTS, sensor response was plotted against concentration with each sensor being represented by a different color/ shape. The R^2 value and equation for each sensor is displayed.

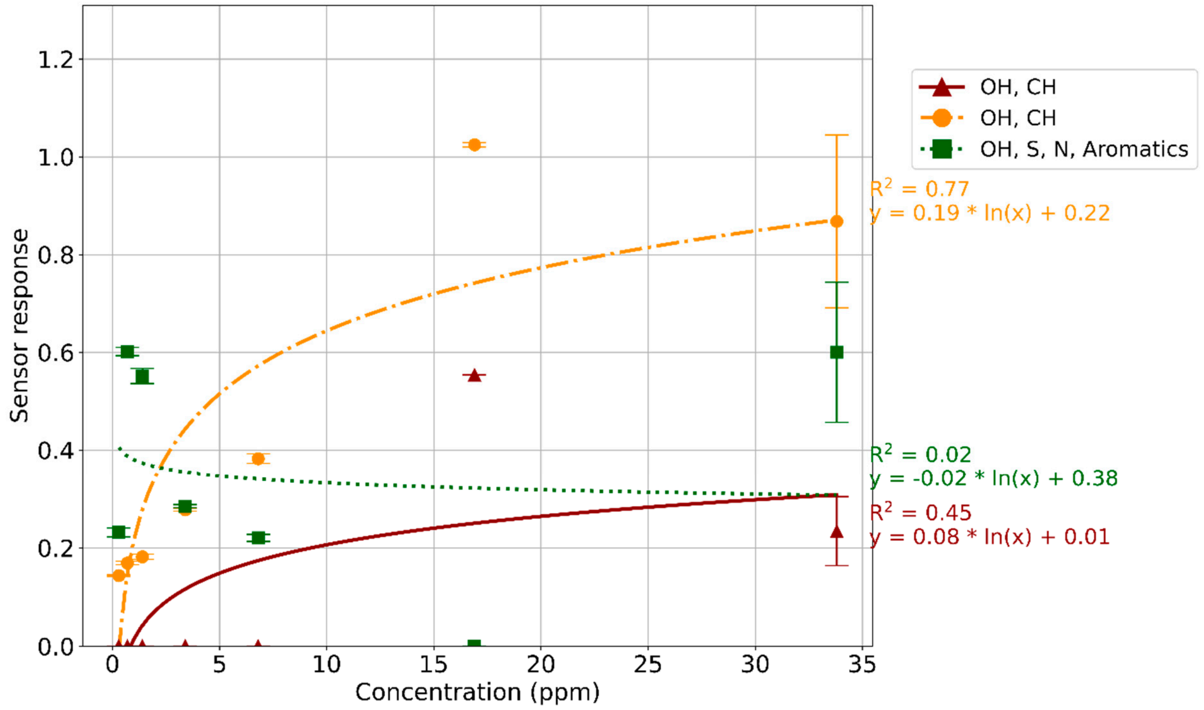

Figure S10: Standard curve of ethylcyclohexane, sensor response was plotted against concentration with each sensor being represented by a different color/ shape. The R^2 value and equation for each sensor is displayed.

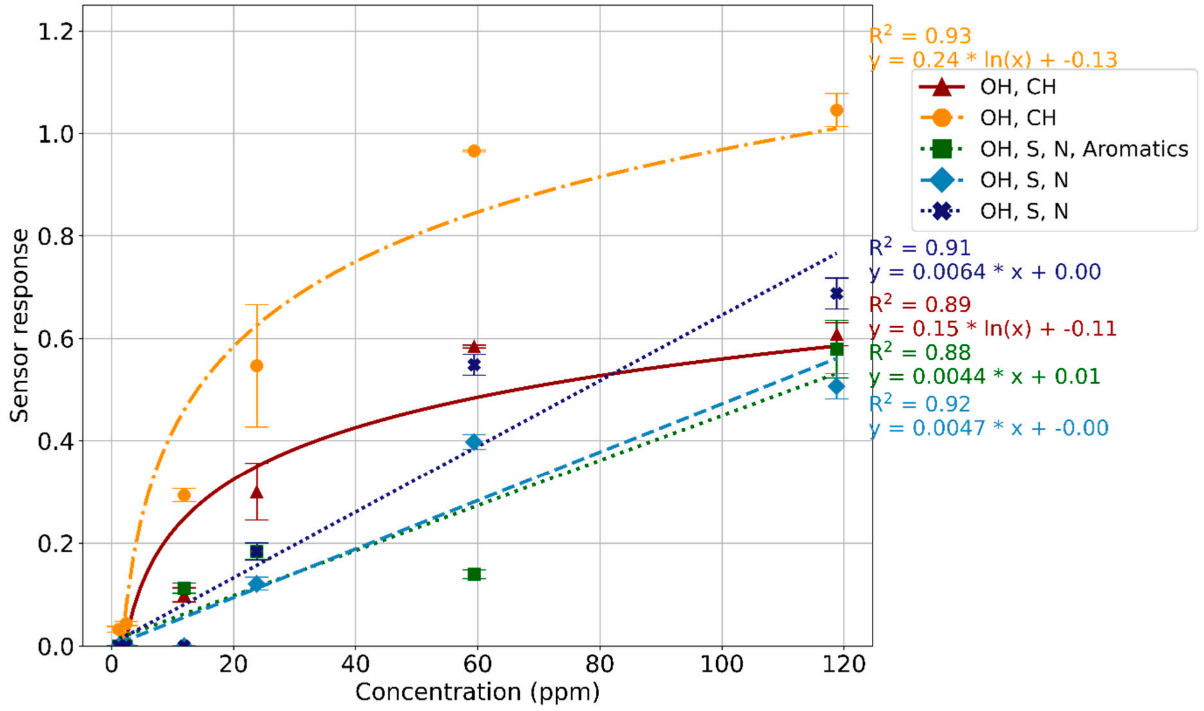

Figure S11: Standard curve of methanol, sensor response was plotted against concentration with each sensor being represented by a different color/ shape. The R^2 value and equation for each sensor is displayed.

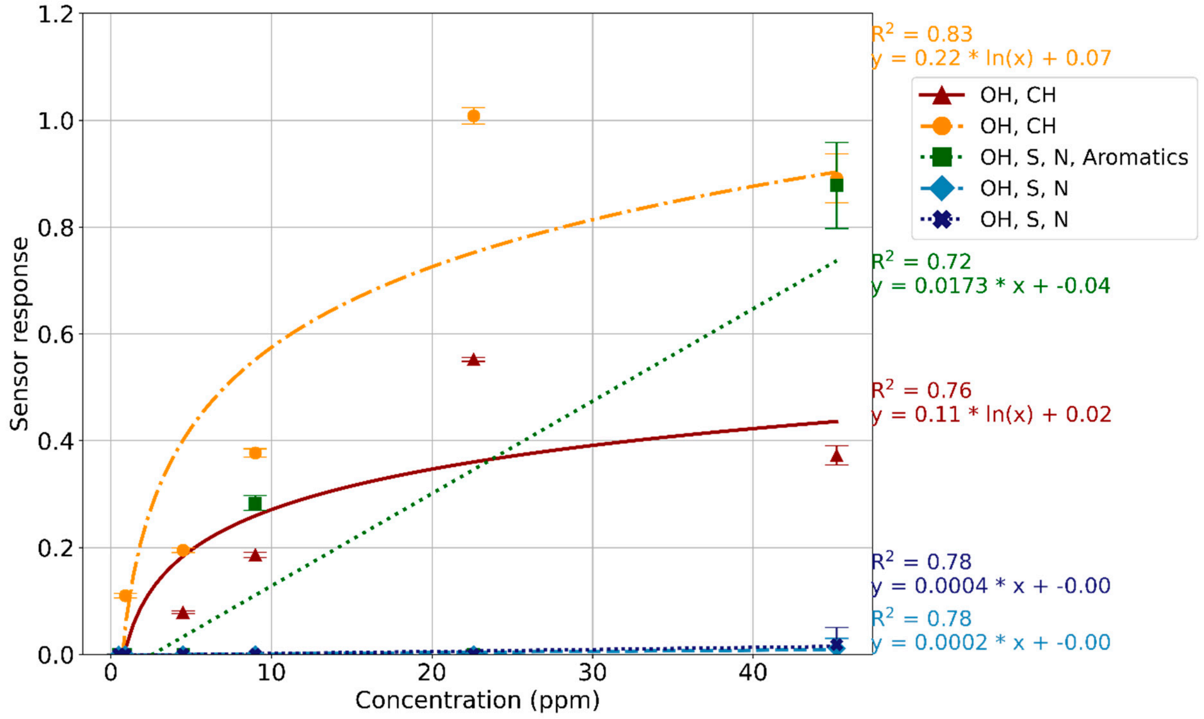

Figure S12: Standard curve of toluene, sensor response was plotted against concentration with each sensor being represented by a different color/ shape. The R^2 value and equation for each sensor is displayed.

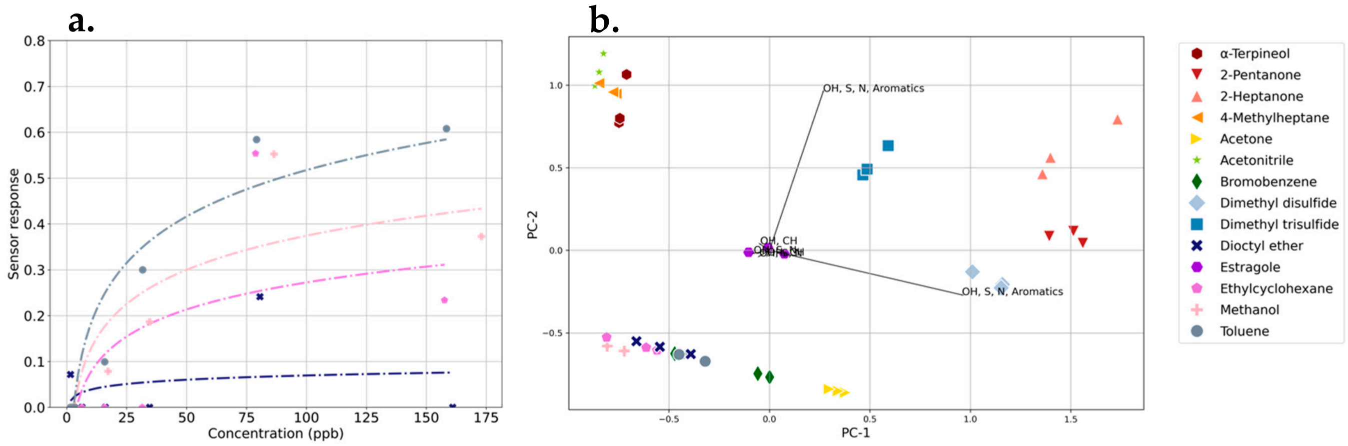

Figure S13: a. Standard curve of methylcyclohexane, toluene and methanol sensor 1 showing the saturation **b.** PCA biplot of the standards in the 16 – 118 ppm range displaying the separation and clustering of all analytes and the contribution of each sensor type to the principal component

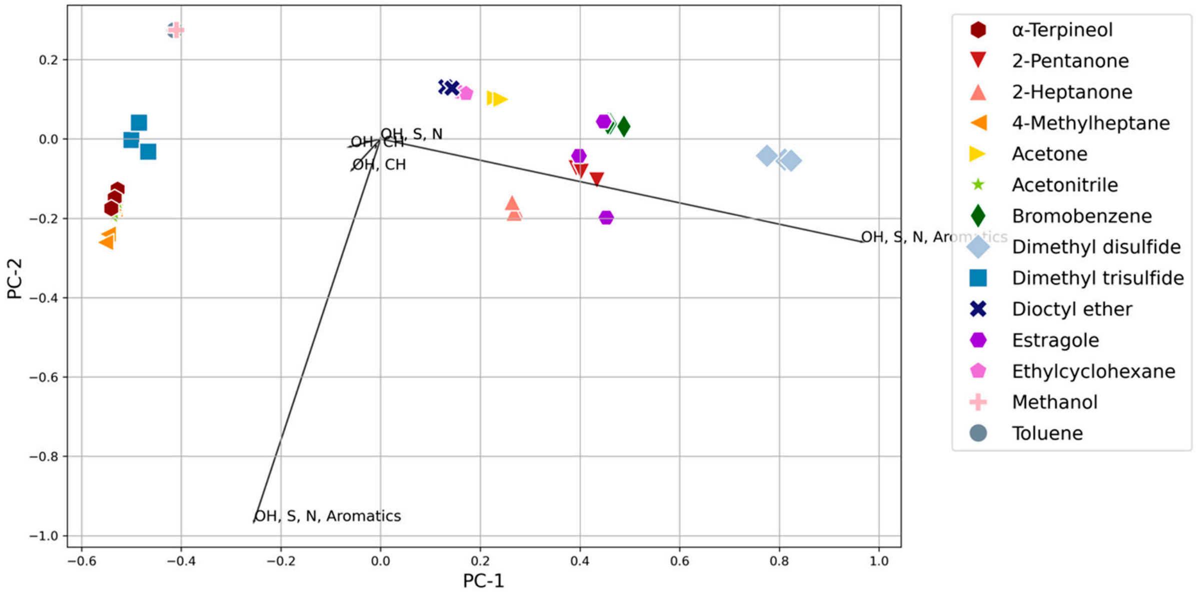

Figure S14: PCA biplot of the standards in the 0.3 – 2.4 ppm range displaying the separation and clustering of all analytes and the contribution of each sensor type to the principal component.

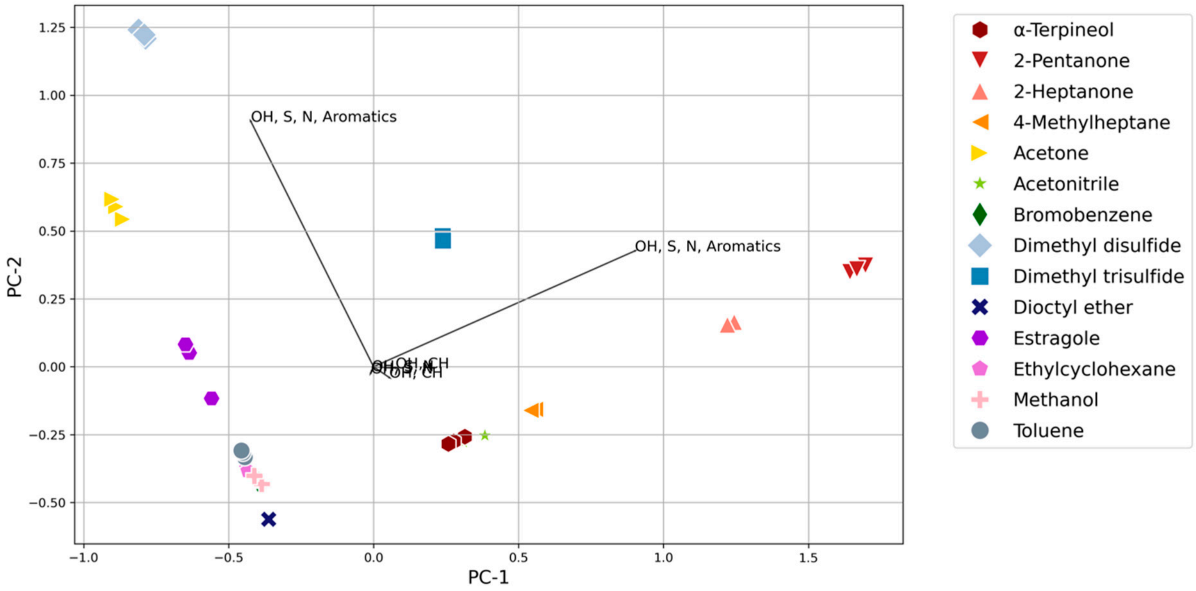

Figure S15: PCA biplot of the standards in the 3.2 – 23.8 ppm range displaying the separation and clustering of all analytes and the contribution of each sensor type to the principal component.

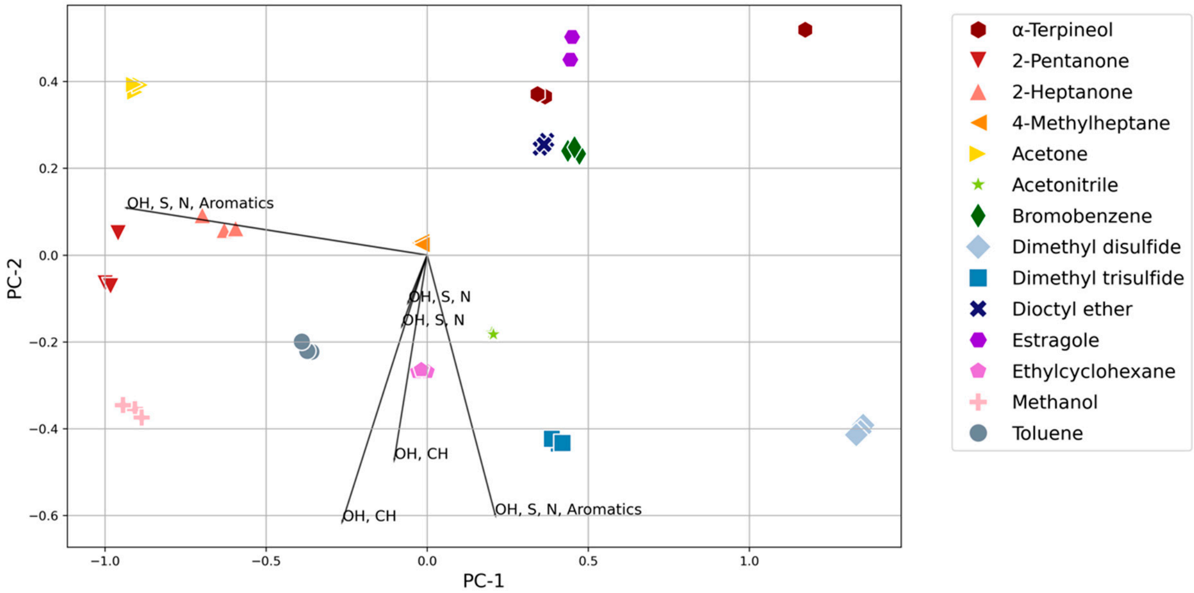

Figure S16: PCA biplot of the standards in the 8 – 59 ppm range displaying the separation and clustering of all analytes and the contribution of each sensor type to the principal component.

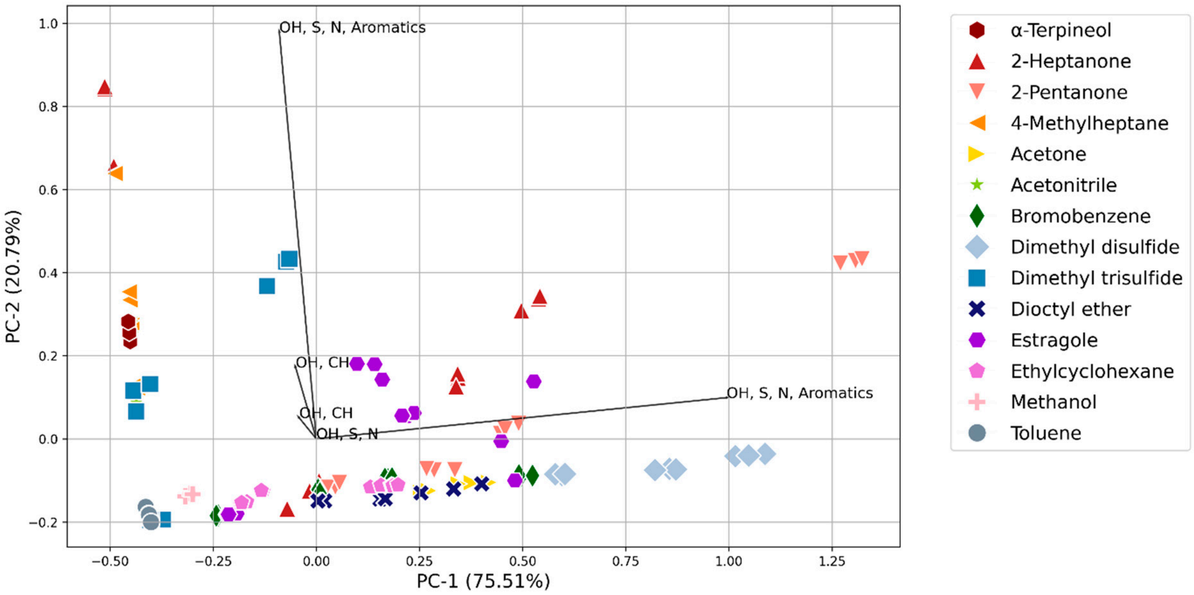

Figure S17: PCA biplot of low concentrations of the analytes tested (0.2 – 5 ppm), displaying the separation and clustering of all analytes and the contribution of each sensor type to the principal component

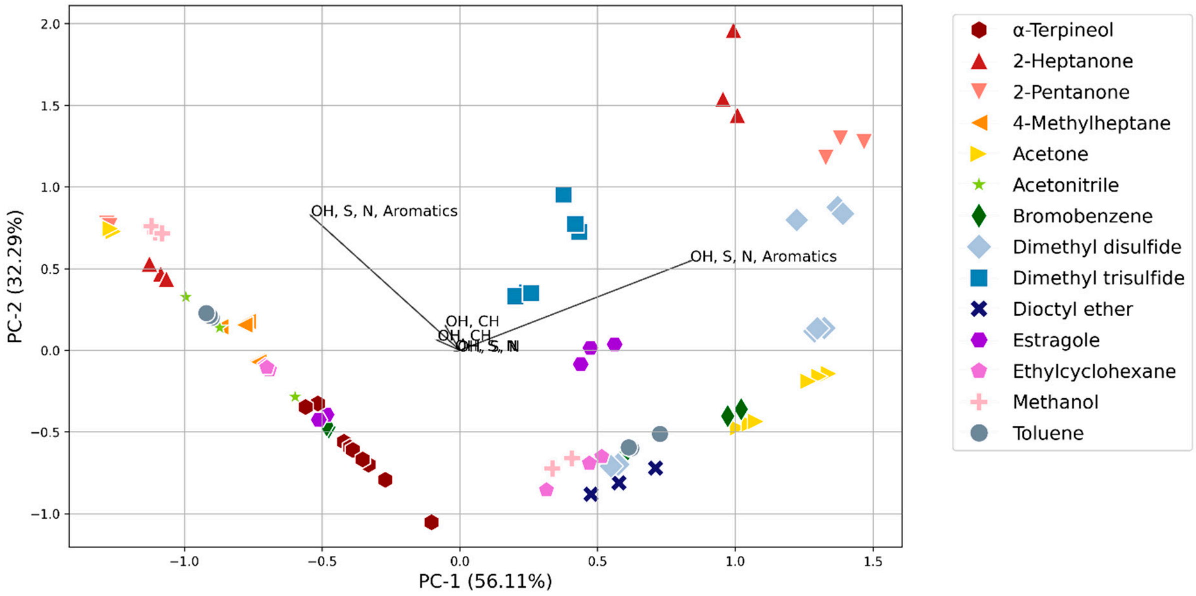

Figure S18: PCA biplot of high concentrations of the analytes tested (10 - 110 ppm), displaying the separation and clustering of all analytes and the contribution of each sensor type to the principal component.

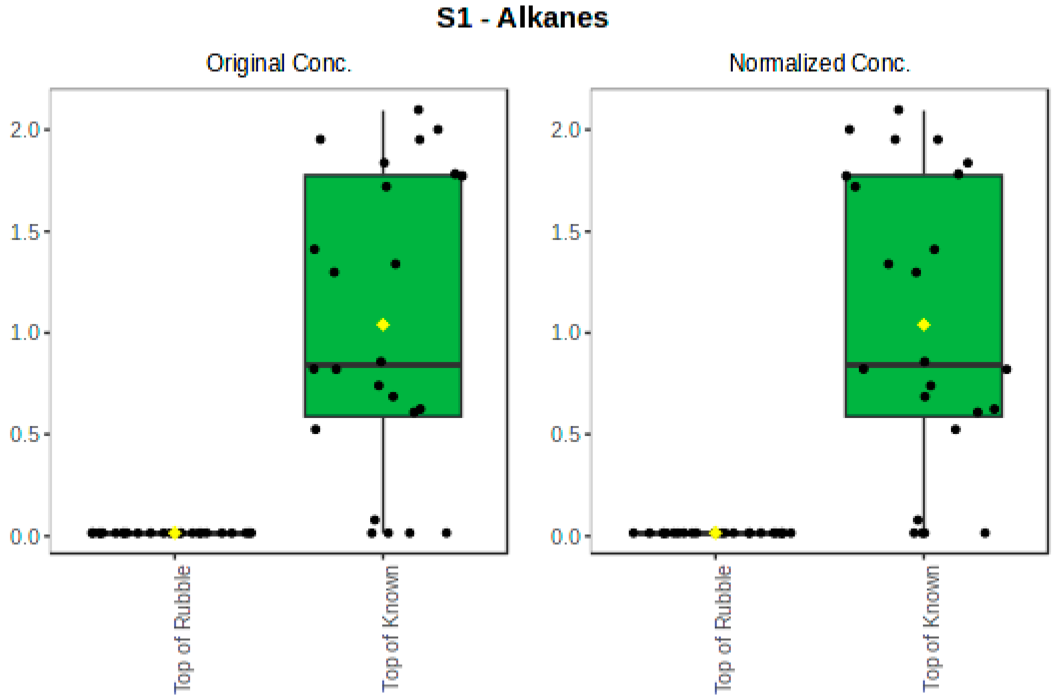

Figure S19: Box and whisker plot for the distribution of Sensor 1 (TGS 2601) response for the control (top of rubble) vs the known (top of known)

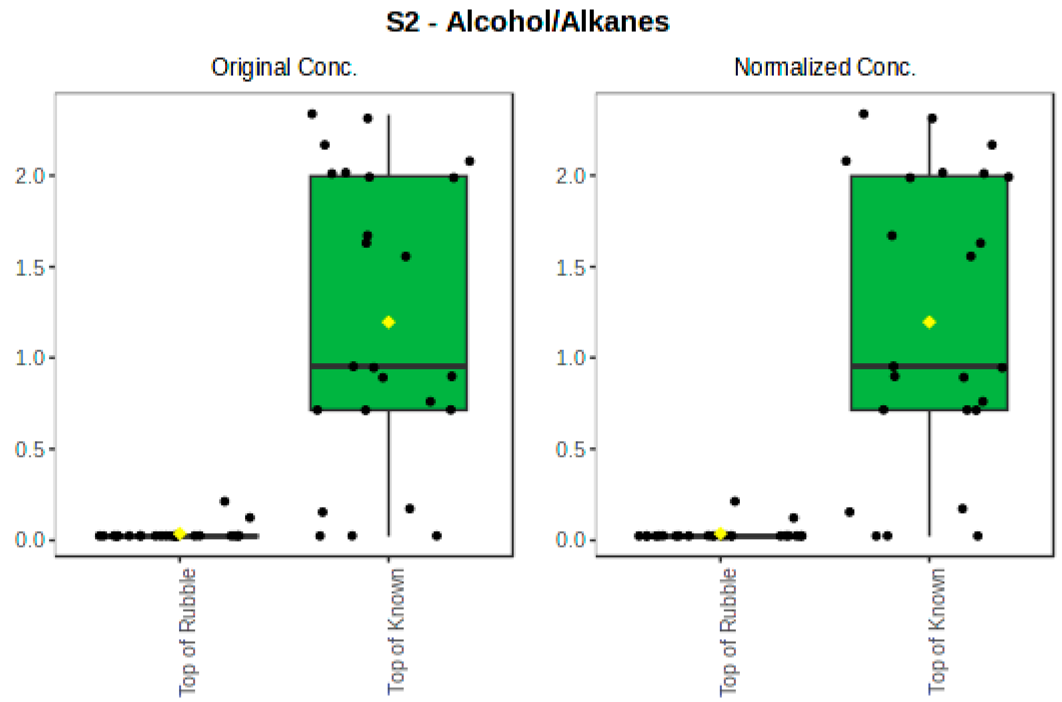

Figure S20: Box and whisker plot for the distribution of Sensor 2 (TGS 2602) response for the control (top of rubble) vs the known (top of known)

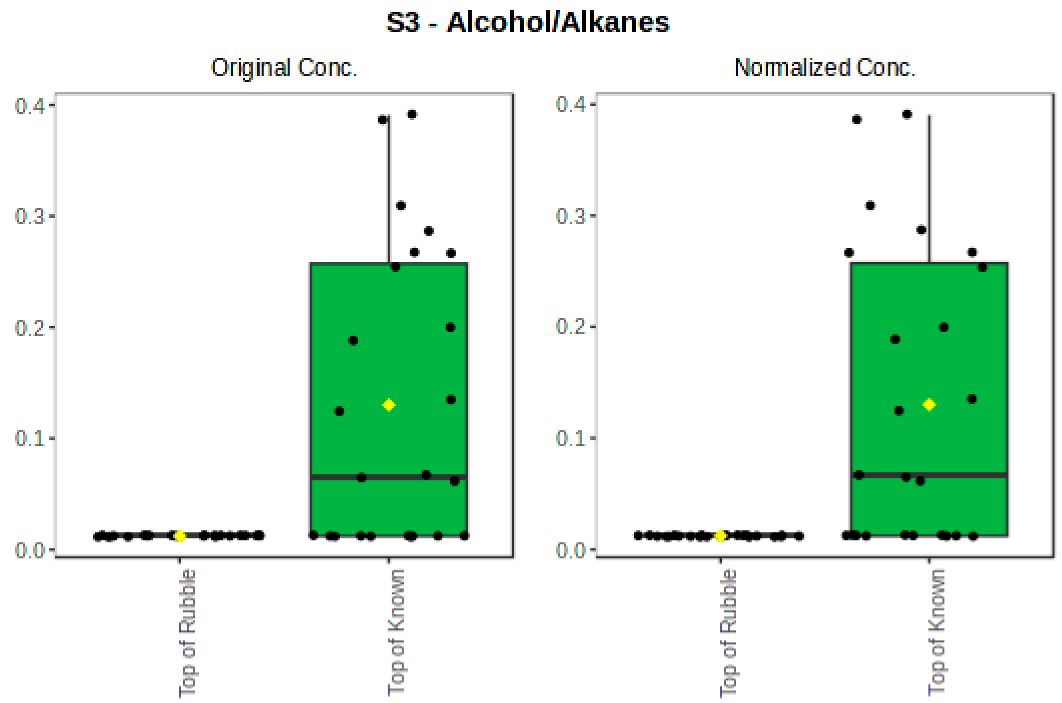

Figure S21: Box and whisker plot for the distribution of Sensor 3 (TGS 2600) response for the control (top of rubble) vs the known (top of known)

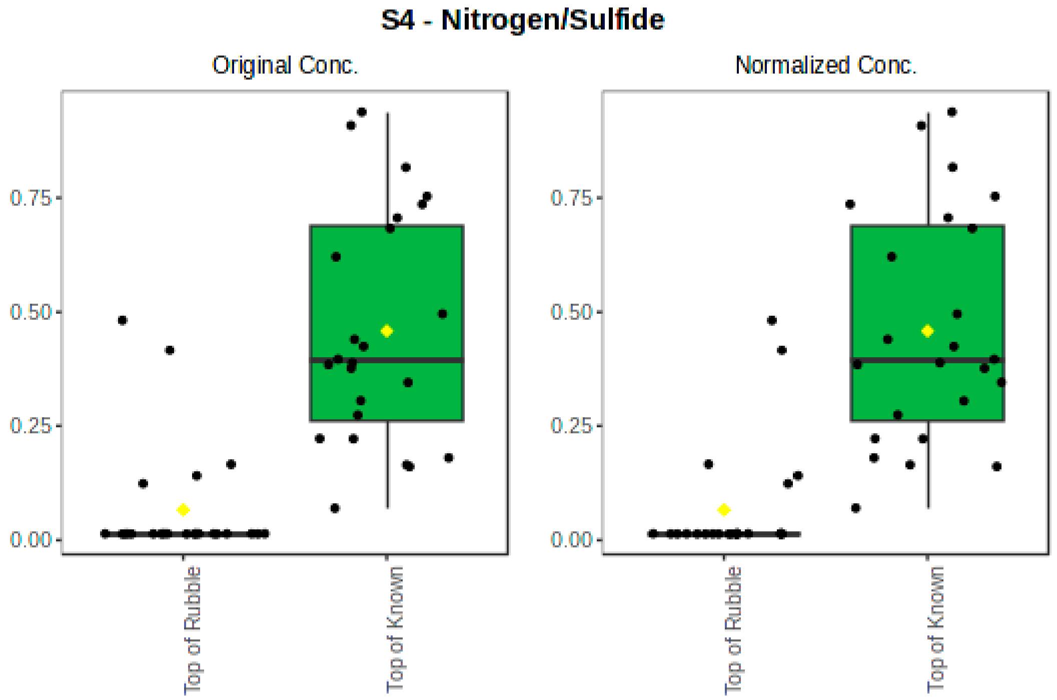

Figure S22: Box and whisker plot for the distribution of Sensor 4 (TGS 2612) response for the control (top of rubble) vs the known (top of known)

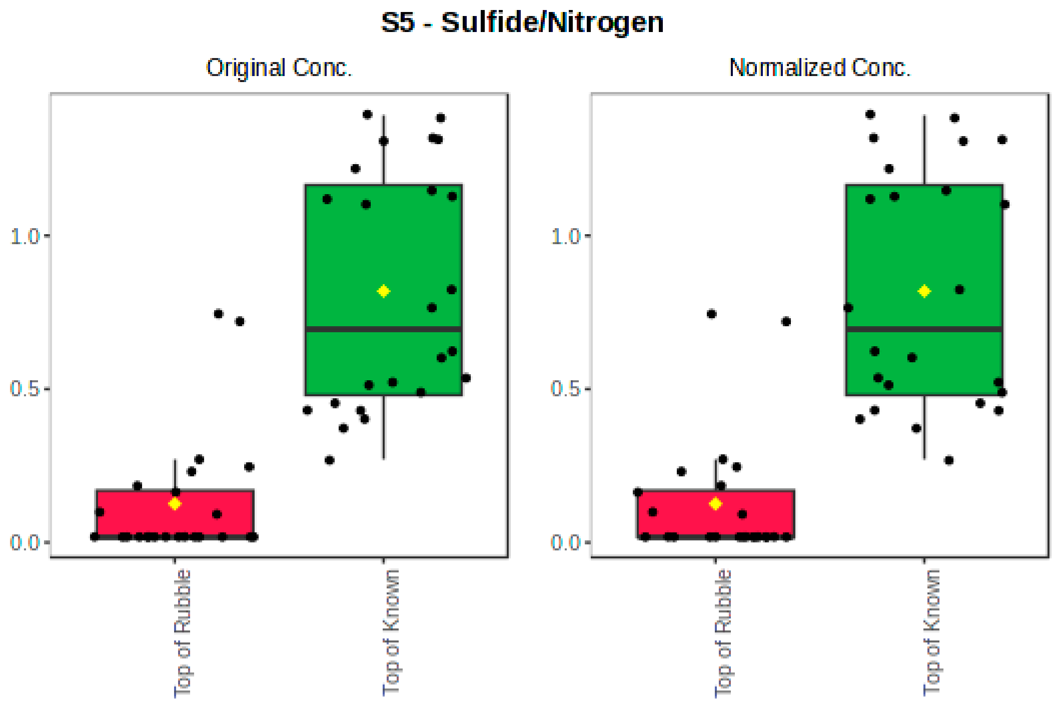

Figure S23: Box and whisker plot for the distribution of Sensor 5 (TGS 2603) response for the control (top of rubble) vs the known (top of known)

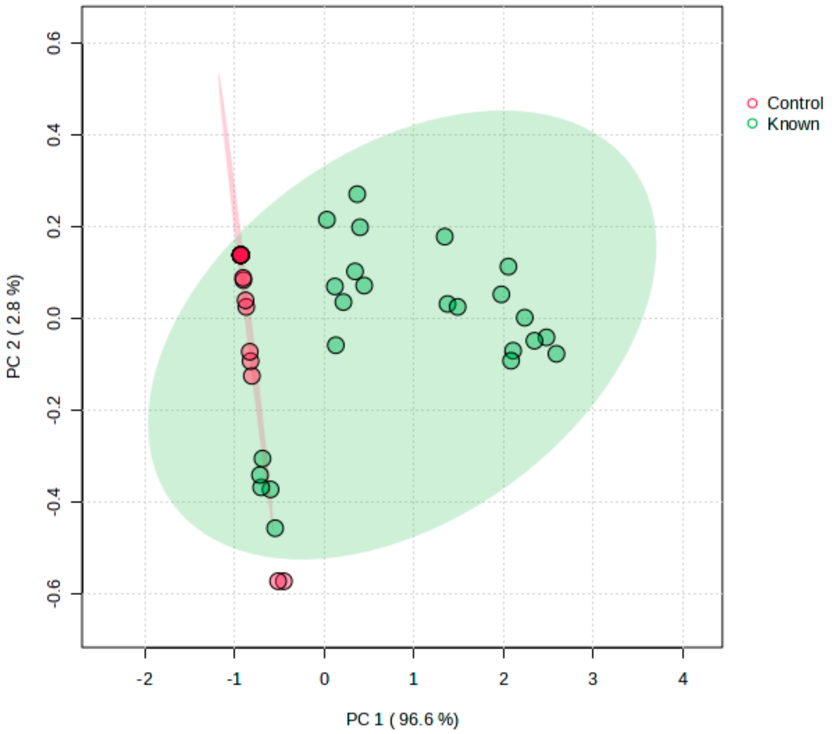

Figure S24: PLS-DA scores plot for the comparison between the sensor response produced from the control (red) and known (green) for each sample taken.

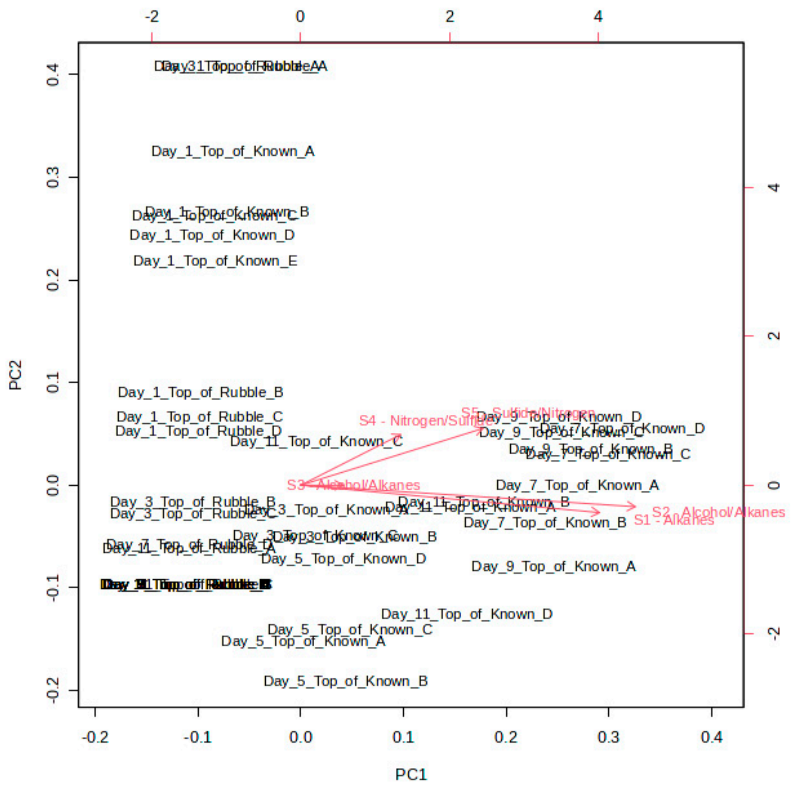

Figure S25: PCA biplot of sensor response, displaying the separation and clustering for each sampling day and replicate, and the contribution of each sensor type to the principal component
